# Infectious Disease Outbreak and Post-Traumatic Stress Symptoms: A Systematic Review and Meta-Analysis

**DOI:** 10.3389/fpsyg.2021.668784

**Published:** 2021-08-05

**Authors:** Dan Qiu, Yilu Li, Ling Li, Jun He, Feiyun Ouyang, Shuiyuan Xiao

**Affiliations:** ^1^Department of Social Medicine and Health Management, Xiangya School of Public Health, Central South University, Changsha, China; ^2^Mental Health Institute, Second Xiangya Hospital, Central South University, Changsha, China

**Keywords:** infectious disease outbreak, systematic review, meta-analysis, COVID-19, post-traumatic stress symptoms

## Abstract

**Background:** As one of the most widely researched consequence of traumatic events, the prevalence of post-traumatic stress symptoms (PTSS) among people exposed to the trauma resulting from infectious disease outbreak varies greatly across studies. This review aimed at examining the pooled prevalence of PTSS among people exposed to the trauma resulting from infectious disease outbreak, summarizing the possible causes of the inconsistencies in the current estimates.

**Methods:** Systematic searches of databases were conducted for literature published on PubMed, EMBASE, Web of Science, the Cochrane Library, PsycArticles, and Chinese National Knowledge Infrastructure (CNKI) until 14 October 2020. Statistical analyses were performed using R software (registration number: CRD42020182366).

**Results:** About 106 studies were included. The results showed that the pooled prevalence of PTSS among the general population exposed to the trauma resulting from infectious disease outbreak was 24.20% (95% CI: 18.54–30.53%), the pooled prevalence of PTSS among healthcare workers was 24.35% (95% CI: 18.38–1.51%), the pooled prevalence of PTSS among patients with infectious disease was 28.83% (95% CI: 18.53–44.86%), and the pooled prevalence of PTSS among suspected cases of infectious disease was 25.04% (95% CI: 18.05–34.73%). Mortality rate was a significant contributor to heterogeneity.

**Conclusions:** Evidence suggests that PTSS were very common among people exposed to the trauma resulting from infectious disease outbreak. Health policymakers should consider both short-term and long-term preventive strategy of PTSS.

## Background

Infectious disease poses a serious threat to public health. Over the past two decades, novel viruses continuing to emerge, the number of reported outbreaks of highly pathogenic or highly transmitted infectious diseases has increased, such as severe acute respiratory syndrome (SARS) in 2003, 2009 influenza A (H1N1) in 2009, and Ebola virus disease (Ebola) in 2014

(Houlihan and Whitworth, 2019). At the end of 2019, a new type of infectious disease emerged, which is known as coronavirus disease 2019 (COVID-19). As of December 10, 2020, over 66.2 million cases of COVID-19 and about 1.5 million deaths have been reported to the WHO (WHO, 2020). The outbreak of infectious disease can spread rapidly, causing enormous losses to individual health, national economy, and social well-being (Steele et al., 2016).

The psychological effects of infectious disease outbreak can be deleterious and far-reaching. Previous research indicates high prevalence rates of clinically relevant post-traumatic stress symptoms (PTSS) among people exposed to the trauma resulting from infectious disease outbreak (such as the outbreak of SARS; Gardner and Moallef, 2015). Patients with post-traumatic stress disorder (PTSD)-related symptoms live under the shadow of past trauma. According to the Diagnostic and Statistics of Mental Disorders, the fifth edition (DSM-5), the clinical features of PTSD include persistent intrusion symptoms, persistent avoidance of stimuli, negative alterations in cognition or mood, and marked alterations in arousal and reactivity, all of which are related to traumatic events (Association, 2013). PTSS could cause clinically significant distress or impairment in social, occupational, or other important areas of functioning (Greene et al., 2016). When an infectious disease breaks out, people may experience many types of psychological trauma, such as directly suffering from the symptoms and traumatic treatment, witness of suffering, and struggling and dying of patients (Fiorillo and Gorwood, 2020). Additionally, individuals may experience the fear of realistic or unrealistic of infection, social isolation, exclusion, and stigmatization, as patients, care and help providers, or even the general public (Kisely et al., 2020; Morganstein and Ursano, 2020). As one of the most widely researched consequence of traumatic events, the prevalence of PTSS among people exposed to the trauma resulting from infectious disease outbreak varies greatly across studies (Lancee et al., 2008; Jung et al., 2020). In order to provide more reliable prevention, it is necessary to determine a more accurate estimation of the prevalence of PTSS among people exposed to the trauma resulting from infectious disease outbreak and to explore the possible causes of the inconsistencies in the current estimates.

Currently, control of the epidemic of COVID-19 is still the dominant task of the whole world, millions of people are scared and even panic of the possible loss of health, life, and wealth (Dutheil et al., 2020). A few epidemic studies reported that experience and witness of the suffering related to COVID-19 resulted in a high prevalence of PTSD-related symptoms (Kisely et al., 2020; Rogers et al., 2020). Although it is too early to predict how many people worldwide will be infected with the virus, it is believed that the numbers of case and death will continue to increase in the following months. Some psychologists draw attention toward PTSD as the second tsunami of the COVID-19 pandemic (Dutheil et al., 2020). For taking effective measures to reduce the psychological sequelae caused by COVID-19 across the world, understanding how infectious disease outbreak cause PTSD and who might be vulnerable are essential. This review aimed at examining the pooled prevalence of PTSS among people exposed to the trauma resulting from infectious disease outbreak (including infectious diseases over the past 20 years and COVID-19), summarizing the possible causes of the inconsistencies in the current estimates, and examining potentially vulnerable populations, try to provide a reference for COVID-19 and possible outbreak of infectious diseases in the future.

## Materials and Methods

This review was reported in accordance with the PRISMA guideline and the Meta-analyses Of Observational Studies in Epidemiology (MOOSE) guidelines (Stroup et al., 2000; Moher et al., 2009). The protocol of this review is registered in the International Prospective Register of Systematic Reviews (registration number: CRD42020182366). See Supplementary Material for the details.

### Search Strategy

PubMed, EMBASE, Web of Science, the Cochrane Library, PsycArticle, and Chinese National Knowledge Infrastructure (CNKI) were independently searched by two reviewers (DQ and YLL), with no restrictions on date or language of publication up until 25 April 2020, and an update search was conducted on 14 October 2020. The following search terms were used: “Infectious disease” (including “infection,” “infectious,” “infectious disease,” “public health emergency,” “public health event,” “SARS,” “Severe Acute Respiratory Syndrome,” “H1N1,” “flu,” “influenza,” “Ebola,” “MERS,” “Middle East Respiratory Syndrome Coronavirus,” “coronavirus,” and “COVID-19”); “Post-traumatic stress disorder” (including “Posttraumatic stress disorder,” “posttraumatic syndrome,” “PTSD,” “stress disorder,” “post-traumatic,” and “post traumatic syndrome”). See Supplementary Table 1 for a full search strategy.

### Study Selection

Studies were included if they meet the following criteria: (1) the study was observational study; (2) information about the prevalence of PTSS among people exposed to the trauma resulting from infectious disease outbreak; (3) the full article was written in English or Chinese; and (4) these outbreaks were SARS, H1N1, H7N9, MERS, Ebola virus disease, Zika virus disease, and COVID-19. Studies were excluded if: (1) the report was a review, comments, meta-analysis, or protocol; (2) the participants with comorbid symptoms or chronic disease (such as mental illness, cancer, etc.); and (3) the report was duplicate results.

### Data Extraction

Two reviewers (DQ and YLL) checked the titles, abstracts, and full texts of the initial search results independently. Data were extracted on first author, year of publication, country or area, type of disease, population, survey period, sample size, response rate, percentage of male participants, average age of participants, instruments used to identify PTSS, prevalence of PTSS, and quality score of the included studies. Any discrepancies that emerged in these procedures were discussed and resolved by involving a third reviewer (SYX).

### Quality Assessment

Two independent reviewers (JH and FYOY) used the established guidelines, the Loney criteria, to evaluate the methodological quality of the included studies, which has been widely used to evaluate observational studies (Loney et al., 1998; Sanderson et al., 2007). The included papers were scored according to eight criteria, such as definition of participants, study design, sampling method, response rate, sample size, and appropriateness of measurement and analysis. The scores range from 0 to 8, with a score of 0–3 as low quality, 4–6 as moderate, and 7–8 as high (Qiu et al., 2020). See Supplementary Table 3 for details on the quality assessment.

### Statistical Analyses

When data were available for three or more studies, the prevalence was combined. When there were 10 or more studies, the quantitative subgroup analysis was conducted. All the statistical analyses were performed using the “meta” (4.12-0) and “metafor” package (2.4-0) of R version 4.0.0. Between-study heterogeneity was evaluated by Cochran's *Q*-test and quantified by the *I*^2^ statistic, with values 50% or more indicating possible heterogeneity (Higgins et al., 2003; Ades et al., 2005). The pooled prevalence of PTSS was combined using the Logit transformation method or Log transformation method by a random effects model if significant heterogeneity was observed across studies (when *p* < 0.05, *I*^2^ > 50%). If more than one dataset was reported for the same group of participants, the outcomes that were assessed at the baseline were used. In order to compare the prevalence from different studies, the subgroup meta-analysis was conducted. Because the subgroup analyses should be interpreted with caution (Jike et al., 2018), we planned *a priori* to limit our subgroup analyses to a small number of baseline characteristics including area, sample size, type of disease, mortality rate of disease, survey time after the outbreak, gender, age, assessment tool, and quality score. The difference between subgroups was examined using the Cochran's *Q* chi-square tests. Mixed-model meta-regression analyses were performed by using the Freeman–Tukey double arcsine method to explore potential moderators on the heterogeneity. Publication bias was investigated by Egger's test. To evaluate the consistency of the results, sensitivity analysis was performed by removing each study individually. All the statistical tests were two-sided, with a significance threshold of *p* < 0.05.

## Results

### Literature Search

**Figure 1:**
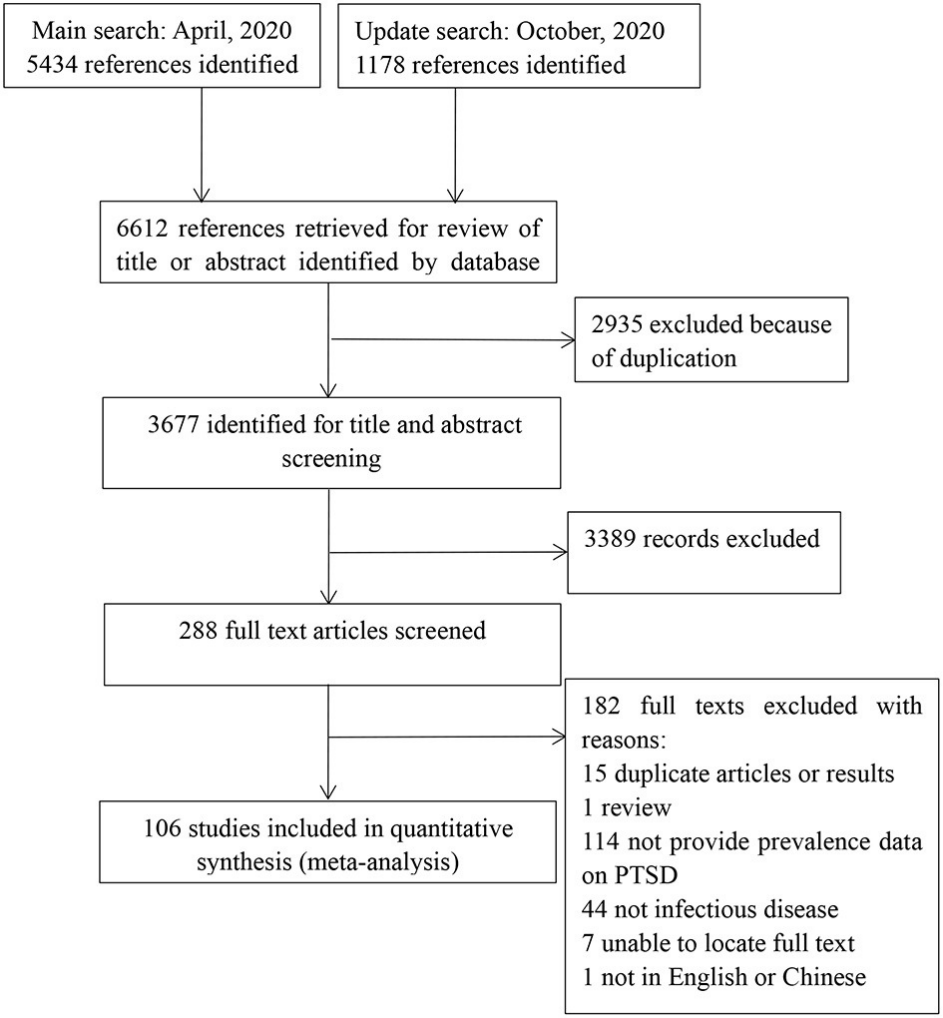
Flow of studies through review.

As shown in Figure 1, a total of 6,612 references were identified. Among them, 2,953 duplicates were removed. By screening titles and abstracts, 3,019 irrelevant articles were excluded. A total of 288 potentially relevant full-text articles were independently assessed based on the selection criteria. Further, 182 studies were excluded because of the following reasons: duplicate articles or results (*n* = 15), review (*n* = 1), did not provide data on PTSS (*n* = 114), not infectious disease (*n* = 44), unable to locate full text (*n* = 7), and not in English or Chinese (*n* = 1). Finally, 106 eligible studies were included in this review. See Figure 1 for the details.

### Study Characteristics

One hundred and six papers met the inclusion criteria. Of the included studies, 78 were of COVID-19 (Alkhamees et al., 2020; Barbato and Thomas, 2020; Blekas et al., 2020; Bo et al., 2020; Caillet et al., 2020; Cai X. et al., 2020; Cai Z. et al., 2020; Cardel et al., 2020; Castelli et al., 2020; Chang and Park, 2020; Chen B. et al., 2020; Chen et al., 2020; Chew et al., 2020; Chi et al., 2020; Civantos et al., 2020a,b; Cortes-Alvarez et al., 2020; Di Tella et al., 2020; Dobson et al., 2020; El-Zoghby et al., 2020; Fekih-Romdhane et al., 2020; Forte et al., 2020; Giusti et al., 2020; Gonzalez Ramirez et al., 2020; Gonzalez-Sanguino et al., 2020; Guo et al., 2020; Hao et al., 2020; Huang et al., 2020; Karatzias et al., 2020; Lahav, 2020; Lai et al., 2020; Lange et al., 2020; Le et al., 2020; Leng, 2020; Leng et al., 2020; Li, 2020; Liang L. et al., 2020; Liang S. W. et al., 2020; Li et al., 2020,a,b; Li G. et al., 2020; Lijun et al., 2020; Liu C. H. et al., 2020; Liu D. et al., 2020; Liu N. et al., 2020; Liu Y. et al., 2020; Li X. C. et al., 2020; Li X. et al., 2020; Luceno-Moreno et al., 2020; Ma et al., 2020; Nie et al., 2020; Qi et al., 2020; Riello et al., 2020; Rodriguez-Rey et al., 2020; Rossi et al., 2020a,b; Seyahi et al., 2020; Sherman et al., 2020; Si et al., 2020; Song et al., 2020; Tan et al., 2020; Tang et al., 2020; Tee et al., 2020; Traunmuller et al., 2020; Varshney et al., 2020; Wang et al., 2020; Wesemann et al., 2020; Xie et al., 2020; Yin et al., 2020; Yuan et al., 2020; Zhang and Ma, 2020a,b; Zhang C. et al., 2020; Zhang et al., 2020; Zhao et al., 2020; Zhou et al., 2020) (Alkhamees et al., 2020; Barbato and Thomas, 2020; Blekas et al., 2020; Bo et al., 2020; Caillet et al., 2020; Cai X. et al., 2020; Cai Z. et al., 2020; Cardel et al., 2020; Castelli et al., 2020; Chang and Park, 2020; Chen B. et al., 2020; Chen et al., 2020; Chew et al., 2020; Chi et al., 2020; Civantos et al., 2020a,b; Cortes-Alvarez et al., 2020; Di Tella et al., 2020; Dobson et al., 2020; El-Zoghby et al., 2020; Fekih-Romdhane et al., 2020; Forte et al., 2020; Giusti et al., 2020; Gonzalez Ramirez et al., 2020; Gonzalez-Sanguino et al., 2020; Guo et al., 2020; Karatzias et al., 2020; Lahav, 2020; Lange et al., 2020; Le et al., 2020; Leng, 2020; Leng et al., 2020; Li, 2020; Liang S. W. et al., 2020; Li et al., 2020a,b; Li G. et al., 2020; Lijun et al., 2020; Liu C. H. et al., 2020; Liu D. et al., 2020; Liu Y. et al., 2020; Li X. C. et al., 2020; Li X. et al., 2020; Luceno-Moreno et al., 2020; Ma et al., 2020; Nie et al., 2020; Qi et al., 2020; Riello et al., 2020; Rodriguez-Rey et al., 2020; Rossi et al., 2020a,b; Seyahi et al., 2020; Sherman et al., 2020; Si et al., 2020; Song et al., 2020; Tan et al., 2020; Tee et al., 2020; Traunmuller et al., 2020; Varshney et al., 2020; Wesemann et al., 2020; Xie et al., 2020; Yuan et al., 2020; Zhang and Ma, 2020a,b; Zhang C. et al., 2020; Zhang et al., 2020; Zhao et al., 2020; Zhou et al., 2020), two of Middle East Respiratory Syndrome (MERS) (Lee et al., 2018; Jung et al., 2020), one of Ebola virus disease (Jalloh et al., 2018), one of H7N9 (Tang et al., 2017), two of H1N1 (Xu et al., 2011; Luyt et al., 2012), and the remaining 22 of SARS (Chan and Huak, 2004; Fang et al., 2004; Hawryluck et al., 2004; Sin and Huak, 2004; Chen et al., 2005; Tie-ying et al., 2005; Wu et al., 2005, 2009; Yong et al., 2005; Hongsheng et al., 2006; Kwek et al., 2006; Lee et al., 2006; Maunder et al., 2006; Zhongguo et al., 2006; Laiqi et al., 2007; Lin et al., 2007; Su et al., 2007; Lancee et al., 2008; Reynolds et al., 2008; Hong et al., 2009; Mak et al., 2010; Sim et al., 2010). Six papers were in Chinese, and the remainder in English. Of these, 93 were cross-sectional studies, nine were longitudinal designs, and four were case control studies. Most of the included studies were from Asia, such as China, Singapore, and South Korea. See Table 1 for the details. From the 106 papers, five (4.72%) studies were rated as high quality, 93 (87.73%) were rated as moderate, and eight (7.55%) were rated as low quality. Details of the methodological quality assessments of all 106 studies are showed in Supplementary Table 3.

**Table 1:** Study characteristics of the included studies.

**References**	**Study design**	**Type of disease**	**Population**	**Event/N**	**Survey time after the outbreak (month)**	**Mean age**	**Percentage of male participants (%)**	**Response rate (%)**	**Assessment tool**	**Quality score**
Chan and Huak (2004) Singapore	CS	SARS	Healthcare workers	127/661	2	/	/	67.0	IES (≥30)	6
Fang et al. (2004) China	CS	SARS	SARS patients	28/286	4	33.4 ± 11.3	47.2	100.0	CIDI	7
Hawryluck et al. (2004) Japan, Canada	CS	SARS	Healthcare workers	35/129	/	/	/	/	IES-R (≥20)	4
Sin and Huak (2004) Singapore	CS	SARS	Healthcare workers	6/47	/	/	/	85.4	IES-R (≥30)	6
Chen et al. (2005) China	CS	SARS	Healthcare workers	14/128	2	27.2 ± 3.6	0.0	100.0	IES (≥35)	5
Wu et al. (2005) Hong Kong	CS	SARS	SARS patients	11/195	1		43.1	41.0	IES-R	3
Tie-ying et al. (2005) China	CS	SARS	SARS patients /Healthcare workers/ General population	2/4 5/128 2/30	9	/	0.0/ 21.1/33.3	/	PCL-C	4
Yong et al. (2005) China	CS	SARS	SARS patients/ General population	65/114 29/93	3	36.9 ± 13.9 34.9 ± 12.3	45.6/ 38.7	100.0/100.0	IES-R (≥20)	5
Zhongguo et al. (2006) China	CS	SARS	SARS patients	65/117	3	36.9 ± 13.9	44.4	100.0	IES-R (≥19)	5
Kwek et al. (2006) Singapore	CS	SARS	SARS patients	26/63	3	34.8 ± 10.4	20.6	40.0	IES (≥26)	6
Hongsheng et al. (2006) China	F	SARS	SARS patients	31/67	3	25.3 ± 8.5	36.8	88.1	CCMD- III	5
Lee et al. (2006) Hong Kong	CS	SARS	General population	13/146	2	/	/	/	IES-R (≥26)	4
Maunder et al. (2006) Toronto, Canada	CS	SARS	Healthcare workers	96/769	13	43 ± 9.5	/	39.0	IES (≥26)	5
Lin et al. (2007) Taiwan	CS	SARS	Healthcare workers	16/92	6	34.0	8.7	100.0	DTS-C (≥40)	6
Laiqi et al. (2007) China	CS	SARS	Healthcare workers	5/56	12	/	/	/	CCMD- III	3
Su et al. (2007) China	F	SARS	Healthcare workers	29/102	3	43.0 ± 9.5	0.0	/	DTS-C (≥23)	5
Lancee et al. (2008) Japan	CS	SARS	Healthcare workers	2/139	24	45.0	13.0	/	DSM-IV	4
Reynolds et al. (2008) Canada	CS	SARS	General population	148/1057	3	49.2 ± 15.7	37.0	55.3	IES-R (≥20)	7
Wu et al. (2009) China	CS	SARS	Healthcare workers	55/549	36	/	23.5	83.0	IES-R (≥20)	7
Hong et al. (2009) China	F	SARS	SARS patients	28/70	2	38.5 ± 12.3	32.9	81.4	CCMD-III	5
Mak et al. (2010) Hong Kong	F	SARS	SARS patients	23/90	30	41.1 ± 12.1	37.8	96.8	DSM-IV	6
Sim et al. (2010) Singapore	CS	SARS	General population	107/415	3	36.6 ± 13.9	59.3	78.0	IES-R	5
Xu et al. (2011) China	CS	H1N1	General population	22/1082	7	20.2	56.3	100.0	PCL-C	4
Luyt et al. (2012) France	CC	H1N1	H1N1 patients	16/40	4	39.0	48.7	100.0	IES (≥26)	5
Tang et al. (2017) China	CS	H7N9	Healthcare workers	21/102	20	/	33.3	/	PCL-C	3
Jalloh et al. (2018) Sierra Leone	CS	Ebola	General population	570/3,564	12	35.0 ± 15.0	50.0	98.0	IES-6	6
Lee et al. (2018) South Korea	F	MERS	Healthcare workers	183/359	2	/	18.1	19.9	IES-R (≥25)	4
Jung et al. (2020) South Korea	CS	MERS	Healthcare workers	84/147	/	/	0.0	49.0	IES-R (≥18)	5
Castelli et al. (2020) Italy	CS	COVID-19	General population	265/1,321	3	35.1 ± 14.0	31.0%	/	PCL-C	3
Zhang C. et al. (2020) China	CS	COVID-19	High school students	222/1,025	3	15.5 ± 1.8	51.5	87.4	IES-R (≥30)	7
Tee et al. (2020) Philippines	CS	COVID-19	General population	316/1,879	3	34.5 ± 13.4	31.0	75.4	IES-R (≥24)	5
Si et al. (2020) China	CS	COVID-19	Healthcare workers	347/863	1	/	29.3	76.0	IES-6 (≥10)	6
Rodriguez-Rey et al. (2020) Spain	CS	COVID-19	General population	1559/3,055	2	32.1 ± 12.9	29.3	/	IES-R (≥24)	5
Nie et al. (2020) China	CS	COVID-19	Healthcare workers	194/263	0.5	/	23.3	96.3	IES-R (≥20)	5
Liang S. W. et al. (2020) China	CS	COVID-19	College students	1822/4,164	1	/	52.0	/	IES-6	6
Li G. et al. (2020) China	CC	COVID-19	Healthcare workers	1382/4,369	0.5	/	0.0	82.2	IES-R (≥34)	7
Giusti et al. (2020) Italy	CS	COVID-19	Healthcare workers	121/330	3	44.6 ± 13.5	37.4	71.2	IES-6 (≥9)	6
Chen B. et al. (2020) China	CS	COVID-19	Healthcare workers / general population	900/1,493	1	/	55.3	93.3	IES-R (≥20)	6
Caillet et al. (2020) France	F	COVID-19	ICU Caregivers	52/208	3	/	25.0	/	IES-R	5
Barbato and Thomas (2020) Italy	CS	COVID-19	General population	33/148	3	41.4 ± 7.1	24.0	40.0	IES-R (≥33)	5
Alkhamees et al. (2020) Saudi Arabia	CS	COVID-19	General population	467/1,160	3	/	36.1	/	IES-R (≥24)	4
Zhou et al. (2020) China	CC	COVID-19	General population	23/859	1	32.7	0.0	/	IES-R (≥33)	5
Zhao et al. (2020) China	CS	COVID-19	General population	29/515	0.25	/	33.6	/	PCL-5	3
Zhang et al. (2020) Taiwan	CS	COVID-19	General population	377/560	1	25.8 ± 2.7	0.0	93.3	: IES-R (≥26)	4
Yin et al. (2020) China	CS	COVID-19	Healthcare workers	15/371	0.5	35.3 ± 9.4	38.5	/	PCL-5 (≥33)	4
Wesemann et al. (2020) Germany	CS	COVID-19	General population	23/60	2	59.0 ± 17.8	53.7	/	PCL-5	3
Wang et al. (2020) China	F	COVID-19	General population	98/1,210	0.25	/	32.7	92.7	IES-R (≥24)	4
Varshney et al. (2020) India	CS	COVID-19	General population	217/653	3	41.8	75.2	/	IES-R (≥24)	4
Traunmuller et al. (2020) Austria	CS	COVID-19	General population	2,377/4,126	3	38.6 ± 13.3	26.0	/	IES-R (≥24)	5
Tang et al. (2020) China	CS	COVID-19	General population	67/2,485	1	19.8	38.3	69.3	PCL-C (≥38)	6
Tan et al. (2020) China	CS	COVID-19	General population	126/673	1	38.8 ± 7.4	74.4	50.8	IES-R (≥18)	5
Song et al. (2020) China	F	COVID-19	Healthcare workers	1,353/14,825	1	34.0 ± 8.2	35.7	/	PCL-C (≥38)	5
Sherman et al. (2020) America	CS	COVID-19	General population	29/591	4	35.9 ± 8.2	22.5	35.3	PCL-5 (≥33)	6
Seyahi et al. (2020) Germany	CS	COVID-19	Hospital workers/ teachers	219/535 132/917	3	42.0/31.0/35.0	46.0/51.0/39.0	42.8/22.3/41.7	IES-R (≥33)	6
Rossi et al. (2020a) Italy	CS	COVID-19	General population	6,604/18,147	3	38.0 ± 23.0	20.5	/	GPS-PTSS	4
Rossi et al. (2020b) Italy	CS	COVID-19	Healthcare workers	681/1,379	3	39.0 ± 16.0	22.8	49.3	GPS-PTSD	6
Riello et al. (2020) Italy	CS	COVID-19	Healthcare workers	433/1,071	4	/	24.6	53.0	IES-R (≥26)	6
Qi et al. (2020) China	CS	COVID-19	COVID-19 patients	5/41	1	40.1 ± 10.1	41.9	52.4	PCL-5 (≥50)	5
Ma et al. (2020) China	CS	COVID-19	General population	164/728	3	32.9 ± 10.4	29.8	72.8	IES-R (≥26)	6
Luceno-Moreno et al. (2020) Spain	CS	COVID-19	Healthcare workers	160/1,422	3	43.8 ± 10.2	13.6	75.3	IES-R (≥20)	6
Liu N. et al. (2020) China	CS	COVID-19	General population	20/285	0.25	/	45.6	95.0	PCL-5 (≥33)	4
Liu D. et al. (2020) China	CS	COVID-19	COVID-19 patients	84/675	2	/	47.0	90.0	PCL-5	6
Liu C. H. et al. (2020) America	CS	COVID-19	General population	285/898	2	24.5	14.1	/	PCL-C (≥38)	5
Li et al. (2020b) China	F	COVID-19	College students	160/1,442	0.5	/	/	71.2	IES-R (≥24)	7
Li et al. (2020a) China	CS	COVID-19	Healthcare workers	640/3,637	0.5	34.4 ± 9.6	37.0	/	IES-R (≥24)	3
Li X. C. et al. (2020) China	CS	COVID-19	Healthcare workers	220/356	0.25	31.3	13.8	98.6	PCL-5	6
Li X. et al. (2020) China	CS	COVID-19	General population	271/398	3	/	50.5	70.2	IES-7	5
Li (2020) China	CS	COVID-19	General population	744/1,109	3	/	56.0	/	IES-R (≥20)	5
Leng et al. (2020) China	CS	COVID-19	Healthcare workers	5/90	2	/	27.8	83.3	PCL-C (≥50)	6
Le et al. (2020) Vietnam	CS	COVID-19	General population	386/1,423	3	35.0	33.4	/	IES-R (≥24)	5
Lange et al. (2020) France	CS	COVID-19	Healthcare workers	23/135	3	47.9 ± 11.4	40.9	31.1	IES-R	5
Lai et al. (2020) China	CS	COVID-19	Healthcare workers	1,017/1,257	0.25	/	23.3	68.7	IES-R (≥26)	6
Lahav (2020) Israel	CS	COVID-19	General population	112/976	3	44.3 ± 14.2	18.4	77.3	PCL-5 (≥33)	5
Karatzias et al. (2020) Ireland	CS	COVID-19	General population	184/1,041	3	/	48.2	/	ITQ	6
Cardel et al. (2020) America	CS	COVID-19	General population	92/250	3	/	15.0	/	IES-6	4
Guo et al. (2020) China	CS	COVID-19	General population	1,944/2,441	0.25	/	47.6	/	PCL-C-2	5
Gonzalez-Sanguino et al. (2020) Spain	CS	COVID-19	General population	550/3,480	2	/	25.0	/	PCL-C	3
Gonzalez Ramirez et al. (2020) Mexico	CS	COVID-19	General population	1,160/3,932	3	33.0	25.5	/	IES-R	4
Forte et al. (2020) Italy	CS	COVID-19	General population	635/2,291	2	30.0 ± 11.5	25.4	/	IES-R(≥33)	5
Fekih-Romdhane et al. (2020) Tunisia	CS	COVID-19	General population	199/603	3	29.2 ± 10.4	26.0	/	IES-R (≥33)	4
El-Zoghby et al. (2020) Egypt	CS	COVID-19	General population	387/510	3	/	34.1	/	IES-R (≥24)	5
Dobson et al. (2020) Australia	CS	COVID-19	Healthcare workers	93/320	3	/	18.4	/	IES-R (≥26)	6
Di Tella et al. (2020) Italy	CS	COVID-19	Healthcare workers	38/145	2	42.9 ± 11.2	27.6	/	PCL-5	3
Cortes-Alvarez et al. (2020) Mexico	CS	COVID-19	General population	555/1,105	3	/	37.9	/	IES-R	6
Civantos et al. (2020b) America	CS	COVID-19	Healthcare workers	210/349	3	/	60.7	/	IES-R (≥26)	6
Civantos et al. (2020a) Brazil	CS	COVID-19	Healthcare workers	43/163	4	/	74.2	23.3	IES-R (≥26)	5
Chi et al. (2020) China	CS	COVID-19	College students	627/2,038	0.75	20.5 ± 1.9	37.0	81.5	PCL-C	5
Chew et al. (2020) Asia-Pacific region	CS	COVID-19	Healthcare workers	91/1,146	3	31.7 ± 7.8	34.9	88.2	IES-R (≥24)	6
Chang and Park (2020) South Korea	CS	COVID-19	COVID-19 patients	13/64	2	54.7 ± 16.6	43.7	58.7	PCL-5 (≥33)	5
Cai Z. et al. (2020) China	CS	COVID-19	Healthcare workers	184/709	0.25	/	3.5	/	IES-R	5
Cai X. et al. (2020) China	CS	COVID-19	COVID-19 patients	39/126	1	45.7 ± 14.0	47.6	100.0	PTSD-SS	4
Bo et al. (2020) China	CS	COVID-19	COVID-19 patients	689/714	2	50.2 ± 12.9	49.1	97.8	PCL-C (≥50)	5
Blekas et al. (2020) Greek	CS	COVID-19	Healthcare workers	45/270	3	37.6 ± 11.9	21.9	/	PSDI-8	4
Zhang and Ma (2020b) China	CS	COVID-19	General population	20/263	0.25	37.7 ± 14.0	40.3	65.7	IES-R	5
Zhang et al. (2020) China	CS	COVID-19	Suspected COVID-19 patients	13/93	1	38.7 ± 13.6	54.8	100.0	PCL-5 (≥33)	6
Lijun et al. (2020) China	CS	COVID-19	Suspected COVID-19 patients	87/306	2	34.8 ± 8.3	7.8	/	PCL-5 (≥38)	4
Yuan et al. (2020) China	CS	COVID-19	Suspected COVID-19 patients	39/126	1	45.7 ± 14.0	47.6	/	PTSD-SS	4
Xie et al. (2020)	CS	COVID-19	General population	72/333	1	31.0 ± 10.1	39.9	93.8	PCL-C (≥40)	4
Liu Y. et al. (2020) China	CS	COVID-19	General population	453/584	1	35.3 ± 8.9	33.0	90.9	PCL-C (≥40)	6
Liu X. et al. (2020) China	CS	COVID-19	Healthcare workers	20/221	2	/	1.0	99.0	PCL-C (≥40)	6
Leng (2020) China	CS	COVID-19	Healthcare workers	24/72	0.25	/	11.1	92.7	IES-R (≥26)	4
Chen et al. (2020) China	CS	COVID-19	Healthcare workers	23/109	1	/	11.9	/	PCL-C (≥38)	6
Hao et al. (2020) China	CC	COVID-19	General population	15/109	1	/	32.9/ 37.6	11.3/81.3	IES-R (≥24)	5
Liang L. et al. (2020) China	CS	COVID-19	General population	84/584	0.5	/	38.1	95.7	PCL-C (≥38)	6
Li et al. (2020) China	CS	COVID-19	Healthcare workers	104/205	0.75	/	14.6	99.9	PCL-C (≥38)	5
Huang et al. (2020) China	CS	COVID-19	Healthcare workers	63/230	0.5	32.6 ± 6.2	18.7	93.5	PTSD-SS (≥55)	6

*CS, cross-sectional study; CC, case–control study; F, follow-up study; COVID-19, coronavirus disease 2019; SARS, severe acute respiratory syndrome; MERS-CoV, Middle East respiratory syndrome; Ebola, Ebola virus disease; DSM-5, Diagnostic and Statistics of Mental Disorders, the fifth edition; H1N1, 2009 influenza A(H1N1); H7N9, H7N9 avian influenza; IES-R, The Impact of Event Scale–Revised; IES-6, The Impact of Event Scale−6; PCL-C, The amended self-reported Posttraumatic Stress Disorder (PTSD) Checklist– Civilian Version; PTSD-SS, post-traumatic stress disorder self-rating scale; PCL-5, the post-traumatic stress disorder checklist-5; ITQ, The International Trauma Questionnaire; PSDI-8, post-traumatic stress disorder−8 inventory*.

### Pooled Prevalence of Post-traumatic Stress Symptoms Among the General Population

**Figure 2:**
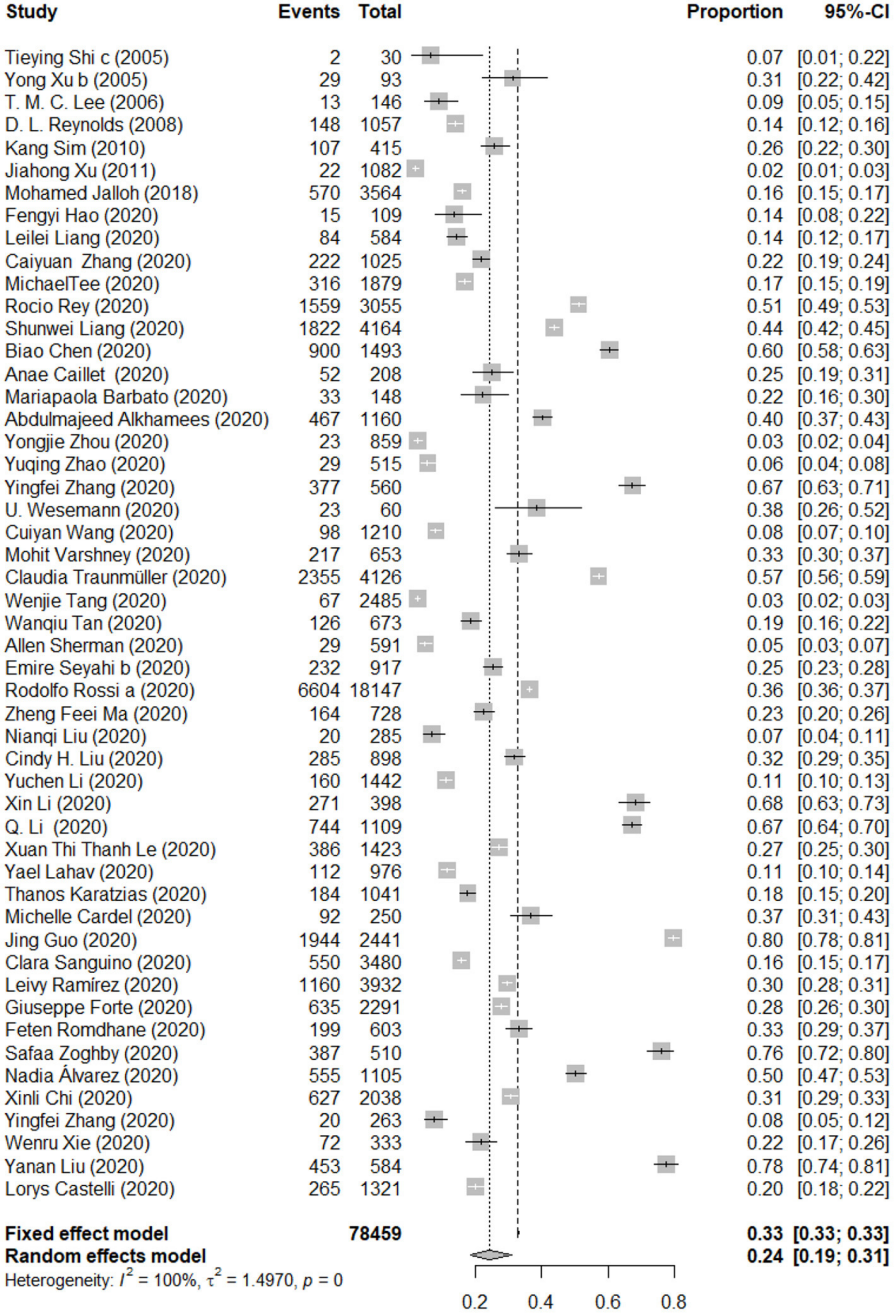
Forest plots of pooled prevalence of post-traumatic stress symptoms among the general population.

There were 51 studies reported the prevalence of PTSS among the general population. The forest plot in Figure 2 depicts the details. A total of 78,459 people exposed to the trauma resulting from an epidemic of infectious disease were identified in the 51 articles, of which 25,826 were reported with PTSS. The random effects model was used to determine the pooled prevalence (*I*^2^ = 99.70%, *p* < 0.001), the pooled prevalence of PTSS among people exposed to the trauma resulting from infectious disease outbreak was 24.20%, with a 95% CI of 18.54–30.53%.

The details of subgroup analyses are presented in Table 2. There were no significant differences in the prevalence of PTSS between age and gender (*Q* = 0.08 and 0.16, *p* > 0.05). Significant difference in the prevalence of PTSS between different types of disease was observed, the pooled prevalence of PTSS among people influenced by COVID-19 was higher than that for people influenced by SARS, Ebola and H1N1 (26.75 vs. 16.42 vs. 15.99 vs. 2.03%; *Q* = 117.12, *p* < 0.05). In addition, a higher mortality rate is associated with a lower prevalence of PTSS (24.39 vs. 15.99%; *Q* = 8.26, *p* < 0.05). The pooled prevalence of PTSS among people in the Eastern Mediterranean region was higher than people in the Western Pacific region, the Southeast Asia region, the America region, the European region, and the Africa region (37.74 vs. 33.23 vs. 29.25 vs. 24.00 vs. 20.78 vs. 15.99%; *Q* = 114.16, *p* < 0.05). Furthermore, there were significant differences in the prevalence of PTSS between different survey time after the outbreak; closer survey time to the point of infectious disease outbreak was associated with a higher prevalence of PTSS (25.96 vs. 5.95%; *Q* = 7.49, *p* < 0.05). There were significant differences in the prevalence of PTSS between studies used different assessment tools (24.44 vs. 14.00%; *Q* = 12.18, *p* < 0.05). In addition, significant difference in the prevalence of PTSS between studies with different quality scores was observed, articles with the highest quality scores showed a high prevalence (12.57 vs. 12.41 vs. 25.86%; *Q* = 19.00, *p* < 0.05). A multivariate meta-regression was carried out to explore the origin of heterogeneity accounted for by the variables, such as type of disease and survey time after the outbreak. However, no significant contributor was found. See **Table 5** for the details.

**Table 2:** Subgroup analysis for the general population.

**Subgroup**	**Studies**	**Pooled prevalence % (95%CI)**	***I*^**2**^ (%)**	**Test of difference within each subgroup**	
				**Q**	***P***
**Mean age**				0.08	0.962
0–30	4	18.97 (4.52–53.64)	99.70		
31–45	25	23.17 (16.08–36.71)	91.20		
>45	2	22.57 (10.67–43.23)	99.70		
**Percentage of male participants (%)**				0.16	0.921
0–33	18	23.52 (16.17–32.90)	98.70		
34–66	28	26.08 (17.44–37.09)	98.50		
67–100	2	25.33 (16.57–36.67)	/		
**Type of disease**				117.12	<0.001
SARS	5	16.42 (9.93–25.95)	92.60		
HIN1	1	2.03 (1.34–3.07)	/		
Ebola	1	15.99 (14.83–17.23)	/		
COVID-19	44	26.75 (20.33–34.32)	99.80		
**Lithality rate**				8.26	0.004
0–20%	50	24.39 (18.60–31.28)	99.70		
>20%	1	15.99 (14.83–17.23)	/		
**WHO region**				114.16	<0.001
Western Pacific	28	20.78 (13.26–31.04)	99.70		
Americas	6	24.00 (12.73–40.61)	99.40		
European	11	29.25 (22.30–37.33)	99.50		
Southeast Asia	1	33.23 (16.34–35.52)	/		
Eastern Mediterranean	4	37.74 (16.62–64.82)	99.40		
Africa	1	15.99 (14.83–17.23)	/		
**Survey time after outbreak (month)**				7.49	0.006
0–6	48	25.96 (20.06–32.89)	99.70		
≥7	3	5.95 (1.91–17.07)	95.40		
**Diagnosis assessment**				12.18	<0.001
Screening tools	50	24.44 (18.65–31.35)	99.80		
Diagnostic tools	1	14.00 (12.04–16.23)	/		
**Sample size**				2.66	0.102
≤ 300	10	17.17 (11.05–25.17)	99.80		
>300	41	26.20 (19.33–34.77)	92.70		
**Quality score**				19.00	<0.001
0–3	3	12.57 (6.73–22.26)	98.30		
4–6	46	12.41 (10.54–14.66)	99.80		
7–8	2	25.86 (19.52–33.40)	57.80		

The results of the Egger's test showed that publication bias was not found in this study (*t* = −2.425, *p* = 0.208). When each study was excluded one by one, the recalculated combined results did not change significantly. The pooled prevalence of PTSS ranged from 23.29% (95% CI: 17.91–29.70%) to 25.13% (95% CI: 19.43–31.85%), and the *I*^2^ statistic varied from 99.70% to 99.80%. The results indicate that no individual study significantly influenced the overall results.

### Pooled Prevalence of Post-traumatic Stress Symptoms Among the Healthcare Workers

**Figure 3:**
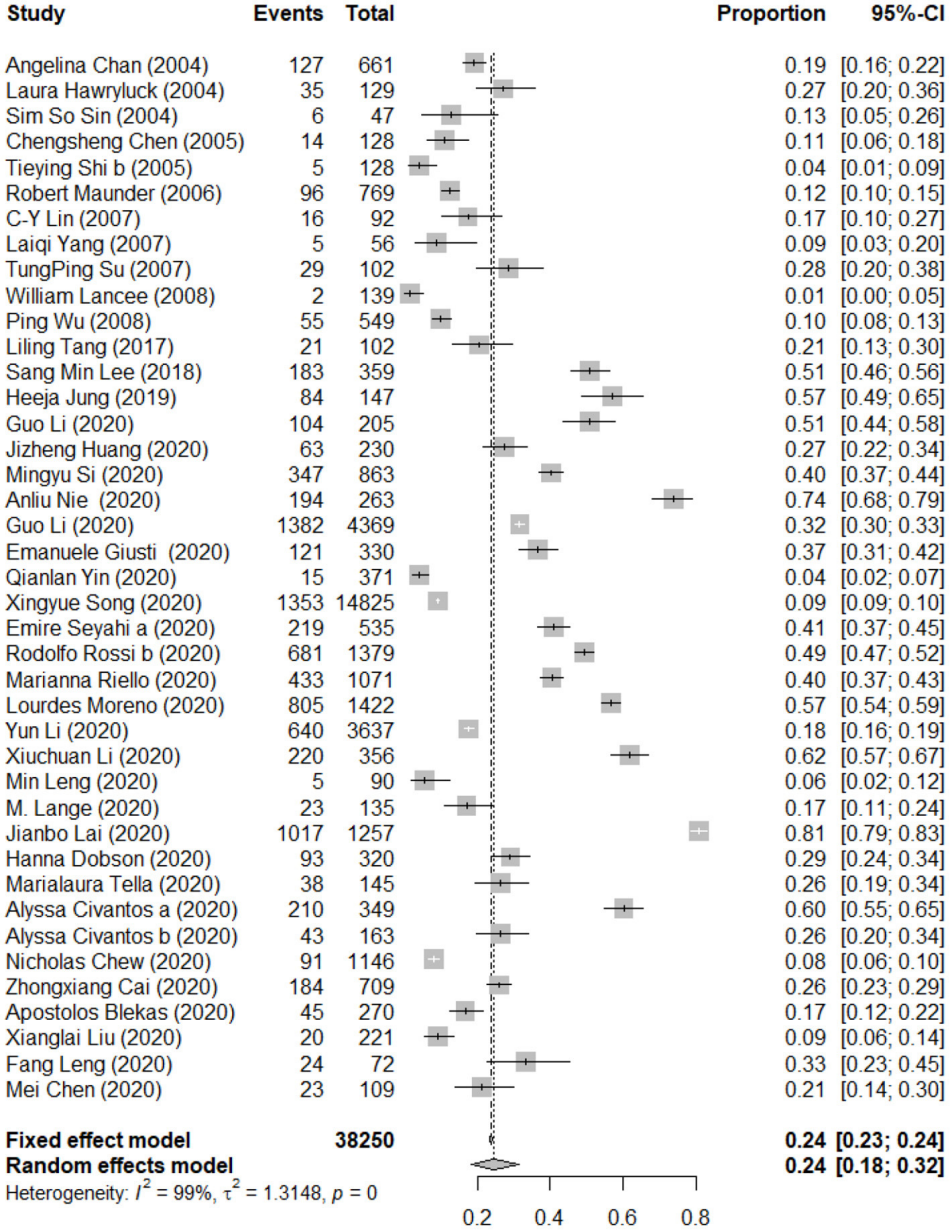
Forest plots of pooled prevalence of post-traumatic stress symptoms among healthcare workers.

A total of 41 studies reported the prevalence of PTSS among the healthcare workers. The forest plot in Figure 3 depicts the details. A total of 38,250 healthcare workers exposed to the trauma resulting from an epidemic of infectious disease were identified in the 41 articles, of which 9,071 were reported with PTSS. The random effects model was used to determine the pooled prevalence (*I*^2^ = 99.40%, *p* < 0.001), the pooled prevalence of PTSS among healthcare workers exposed to the trauma resulting from infectious disease outbreak was 24.35%, with a 95% CI of 18.38–31.51%.

The details of subgroup analyses are presented in Table 3. There were no significant differences in the prevalence of PTSS between age, gender, mortality rate of disease, sample size, and quality score (*Q* = 0.21, 0.19, 3.78, 2.54, and 4.65, *p* > 0.05). Significant difference in the prevalence of PTSS between different types of disease was observed, and the pooled prevalence of PTSS among people influenced by MERS was higher than that for the people influenced by COVID-19, H7N9, and SARS (52.77 vs. 29.64 vs. 20.59 vs. 11.80%; *Q* = 351.95, *p* < 0.05). In addition, a higher mortality rate is associated with a higher prevalence of PTSS (23.19 vs. 42.04%; *Q* = 3.78, *p* < 0.05). The pooled prevalence of PTSS among people in the European region was higher than people in the America region, the Western Pacific region, and the Southeast Asia region (34.47 vs. 29.10 vs. 21.70 vs. 7.94%; *Q* = 70.59, *p* < 0.05). Furthermore, there were significant differences in the prevalence of PTSS between different survey time after the outbreak, and closer survey time to the point of infectious disease outbreak is associated with a higher prevalence of PTSS (29.04 vs. 10.42%; *Q* = 10.09, *p* < 0.05). There were significant differences in the prevalence of PTSS between studies used different assessment tools (24.87 vs. 8.93%; *Q* = 5.84, *p* < 0.05). A multivariate meta-regression was carried out to explore the origin of heterogeneity accounted for by the variables including type of disease, mortality rate, survey time after the outbreak, age, gender, quality score, and sample size. The results of meta-regression showed that mortality rate of disease was a significant contributor to heterogeneity (accounted for 16.81% of the heterogeneity). See **Table 5** for the details.

**Table 3:** Subgroup analysis for healthcare workers.

**Subgroup**	**Studies**	**Pooled prevalence % (95%CI)**	***I*^**2**^ (%)**	**Test of difference within each subgroup**	
				***Q***	***P***
**Mean age**				0.21	0.900
0–30	2	18.22 (9.20–32.88)	99.30		
31–45	14	19.80 (11.36–32.24)	99.60		
>45	1	17.04 (11.59–24.34)	/		
**Percentage of male participants (%)**				1.19	0.551
0–33	27	28.01 (19.72–38.29)	99.30		
34–66	8	19.15 (9.78–34.10)	99.60		
67–100	1	26.38 (20.19–33.67)	/		
**Type of disease**				351.95	<0.001
SARS	11	11.80 (7.59–17.91)	77.53		
H7N9	1	20.59 (13.83–25.93)	/		
MERS	2	52.77 (48.41–57.08)	0.00		
COVID-19	27	29.64 (21.68–39.04)	95.50		
**Lithality rate**				3.78	0.049
0–20%	38	23.19 (17.21–30.49)	99.40		
>20%	3	42.04 (24.57–61.77)	94.60		
**WHO region**				70.59	<0.001
Western Pacific	27	21.70 (14.45–31.25)	94.00		
Americas	5	29.10 (17.30–44.60)	98.00		
European	8	34.47 (25.22–45.08)	98.60		
Southeast Asia	1	7.94 (6.51–9.52)	42.70		
**Survey time after outbreak (month)**				10.09	0.001
0–6	30	29.04 (21.65–37.73)	99.50		
≥7	8	10.42 (5.81–18.00)	93.60		
**Diagnosis assessment**				5.84	0.015
Screening tools	40	24.87 (18.75–32.20)	99.40		
Diagnostic tools	1	8.93 (3.77–19.72)	/		
**Sample size**				2.74	0.098
≤300	21	19.40 (12.93–28.06)	96.20		
>300	20	30.19 (20.81–41.58)	99.70		
**Quality score**				4.65	0.097
0–3	4	17.87 (16.70–19.10)	76.00		
4–6	35	25.57(18.61–34.05)	98.60		
7–8	2	18.64 (7.83–38.19)	80.50		

The results of the Egger's test showed that publication bias was not found in this study (*t* = 0.728, *p* = 0.470). When each study was excluded one by one, the recalculated combined results did not change significantly. The pooled prevalence of PTSS ranged from 23.22% (95% CI: 17.69–29.84%) to 25.62% (95% CI: 19.68–32.62%), and the *I*^2^ statistic varied from 99.20 to 99.40%. The results indicate that no individual study significantly influenced the overall results.

### Pooled Prevalence of Post-traumatic Stress Symptoms Among Patients With Infectious Disease

**Figure 4:**
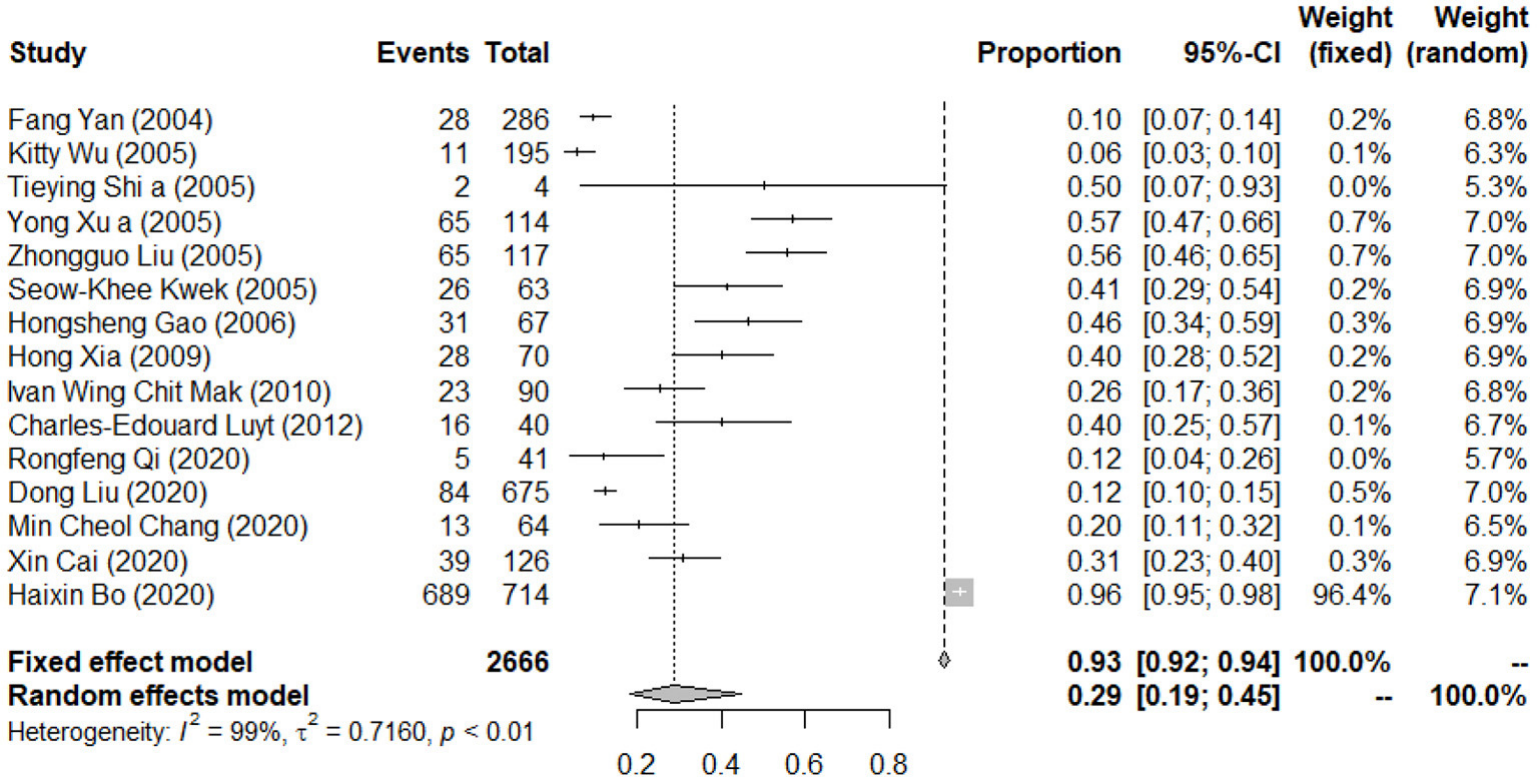
Forest plots of pooled prevalence of post-traumatic stress symptoms among patients with infectious disease.

A total of 15 studies reported the prevalence of PTSS among the patients. The forest plot in Figure 4 depicts the details. A total of 2,666 patients with infectious disease were identified in the 15 articles, of which 1,125 were reported with PTSS. The random effects model was used to determine the pooled prevalence (*I*^2^ = 98.60%, *p* < 0.001), and the pooled prevalence of PTSS among patients with infectious disease was 28.83%, with a 95% CI of 18.53–44.86%.

The details of subgroup analyses are presented in Table 4. There were no significant differences in the prevalence of PTSS between age, gender, type of disease, region, survey time after outbreak, diagnosis tool, sample size, and quality score (*p* > 0.05). A significant difference in the prevalence of PTSS between studies with different quality scores was observed (5.64 vs. 35.45 vs. 9.79%; *Q* = 31.65, *p* < 0.05). A multivariate meta-regression was carried out to explore the origin of heterogeneity accounted for by the variables, such as type of disease and survey time after the outbreak. However, no significant contributor was found. See Table 5 for the details.

**Table 4:** Subgroup analysis for patients with infectious disease.

**Subgroup**	**Studies**	**Pooled prevalence % (95%CI)**	***I*^**2**^ (%)**	**Test of difference within each subgroup**	
				***Q***	***P***
**Mean age**				2.67	0.263
0–30	1	46.27 (35.75–59.89)	/		
31–45	8	31.55 (21.56–46.17)	93.50		
>45	3	40.04 (14.69–99.99)	98.20		
**Percentage of male participants (%)**				3.53	0.060
0–33	4	42.90 (36.60–50.27)	0.00		
34–66	11	24.80 (14.31–42.96)	98.60		
67–100	/				
**Type of disease**				1.36	0.506
SARS	9	30.04 (20.17–44.76)	94.50		
HIN1	1	40.00 (27.37–58.46)	/		
COVID-19	5	25.13 (8.34–75.69)	99.30		
**WHO region**				1.33	0.249
Western Pacific	14	28.15 (17.74–44.67)	99.70		
Americas	/				
European	1	30.33 (22.80–39.08)	99.50		
Southeast Asia	/				
Eastern Mediterranean	/				
Africa	/				
**Survey time after outbreak (month)**				0.04	0.840
0–6	13	28.16 (17.59–45.09)	98.80		
≥7	2	30.40 (17.09–54.07)	37.30		
**Diagnosis assessment**				0.09	0.758
Screening tools	11	26.28 (13.63–50.65)	98.70		
Diagnostic tools	4	14.00 (12.04–16.23)	94.50		
**Sample size**				0.69	0.407
≤300	13	29.29 (19.65–41.24)			
>300	2	66.47 (4.82–98.37)			
**Quality score**				31.65	<0.001
0–3	1	5.64 (3.18–10.02)	/		
4–6	13	35.45 (23.11–54.37)	98.50		
7–8	1	9.79 (6.89–13.02)	/		

**Table 5:** Meta-regression analysis for the included studies.

**Group**	***β***	**95% CI**		***P***	***R^**2**^***
		**Lower**	**Upper**		
**Healthcare workers**					16.81%
**Area** (Western Pacific vs. others)	0.04	−0.14	0.24	0.634	
**Mortality rate** (0–20% vs. >20%)	0.63	0.13	1.14	0.012	
**Type of disease** (coronavirus infections^a^ vs. others)	−0.16	−0.35	0.01	0.069	
**Survey time after the outbreak** (0–6 vs. >6 month)	−0.04	−0.24	0.14	0.638	
**Quality score** (0–3 vs. 4–6 vs.7–8)					
0–3 (reference)					
4–6	0.16	−0.07	0.40	0.169	
7–8	0.06	−0.33	0.46	0.747	
**Sample size** (0–300 vs. >300)	0.11	−0.03	0.26	0.126	
**General population**					9.65%
**Area** (Western Pacific vs. others)	0.02	−0.02	0.07	0.384	
**Mortality rate** (0–20% vs. >20%)	0.14	−0.56	0.85	0.682	
**Type of disease** (coronavirus infections vs. others)	−0.29	−0.99	0.41	0.416	
**Survey time after the outbreak** (0–6 vs. > 6 month)	−0.15	−0.67	0.36	0.551	
**Quality** score (0–3 vs. 4–6 vs.7–8)					
0–3 (reference)					
4–6	0.29	−0.04	0.62	0.090	
7–8	0.04	−0.41	0.51	0.837	
**Sample size** (0–300 vs. >300)	0.14	−0.02	0.32	0.098	
**Patients with infectious disease** ^b^					0.00%
**Area** (Western Pacific vs. others)	−0.02	−0.65	0.59	0.931	
**Survey time after the outbreak** (0–6 vs. >6, month)	−0.02	−0.24	0.66	0.361	
**Quality score** (0–3 vs. 4–6 vs.7–8)					
0–3 (reference)					
4–6	0.41	−0.19	1.02	0.183	
7–8	0.07	−0.73	0.88	0.854	
**Sample size** (0–300 vs. >300)	0.27	−0.52	0.47	0.907	

a *This group include SARS, MERS, and COVID-19*.

b *Type of disease dropped out from the model*.

The results of the Egger's test showed that publication bias was not found in this study (*t* = −6.138, *p* = 3.553). When each study was excluded one by one, the recalculated combined results did not change significantly. The pooled prevalence of PTSS ranged from 23.22% (95% CI: 17.69–29.84%) to 32.23% (95% CI: 20.75–50.05%), and the *I*^2^ statistic varied from 95.40 to 98.7%. The results indicate that no individual study significantly influenced the overall results.

### Pooled Prevalence of Post-traumatic Stress Symptoms Among the Suspected Cases of Infectious Disease

**Figure 5:**
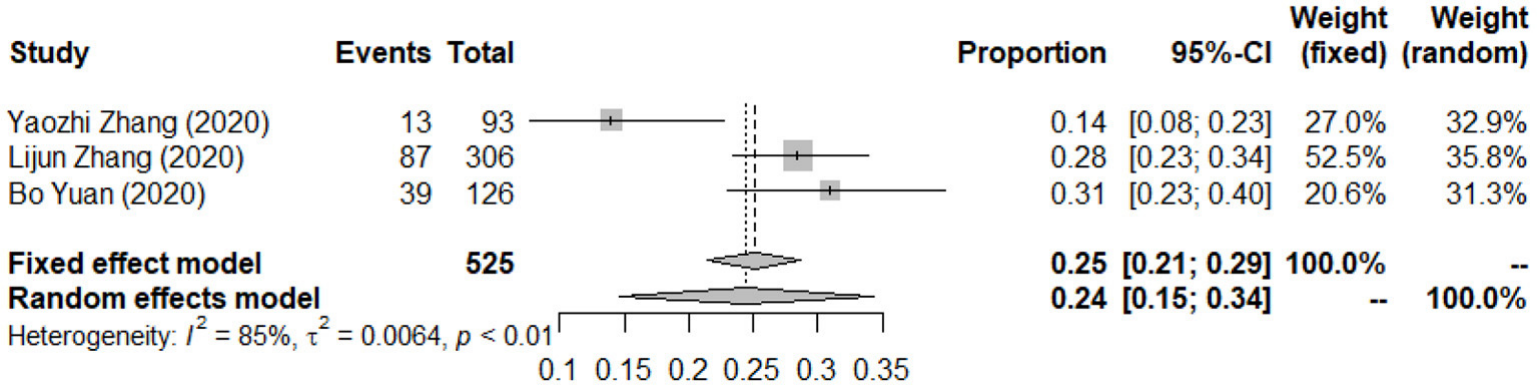
Forest plots of pooled prevalence of post-traumatic stress symptoms among suspected cases.

A total of three studies reported the prevalence of PTSS among the suspected cases. The forest plot in Figure 5 depicts the details. A total of 525 suspected cases of infectious disease exposed to the trauma resulting from an epidemic of infectious disease were identified in the three articles, of which 139 were reported with PTSS. The random effects model was used to determine the pooled prevalence (*I*^2^ = 74.50%, *p* < 0.001), the pooled prevalence of PTSS among suspected cases exposed to the trauma resulting from infectious disease outbreak was 25.04%, with a 95% CI of 18.05–34.73%.

## Discussion

### Key Findings

This review has highlighted the importance of considering the psychological impacts of people exposed to the trauma resulting from infectious disease outbreak. The results showed that the pooled prevalence of PTSS among the general population was 24.20% (95% CI: 18.54–30.53%), the pooled prevalence of PTSS among the healthcare workers was 24.35% (95% CI: 18.38–31.51%), the pooled prevalence of PTSS among patients with infectious disease was 28.83% (95% CI: 18.53–44.86%), and the pooled prevalence of PTSS among the suspected cases of infectious disease was 25.04% (95% CI: 18.05–34.73%), and several factors including type of disease, mortality rate of disease, region, survey time after outbreak, assessment tool, sample size, and quality score were associated with PTSS. Mortality rate of disease was a significant moderator for heterogeneity. Further research is needed to identify effective strategies for preventing and treating PTSS among people exposed to the trauma resulting from infectious disease outbreak.

### Comparison With the Literature

The pooled prevalence of PTSS among different population exposed to the trauma resulting from infectious disease outbreak in this study ranged from 24.20 to 28.83%, which was higher than flood survivors (15.74%) and hurricane survivors (Liu et al., 2017; Wang et al., 2019), but similar to earthquake survivors (Dai et al., 2016) and civilian war survivors (23.66–26.00%) (Morina et al., 2018). Compared with infectious diseases, some natural disasters, such as flood and hurricane, can be predicted, whereas earthquakes and infectious disease were often happened suddenly and without a warning and pose a huge threat to health and property of people in a short period of time (Dai et al., 2016). Therefore, earthquakes might have caused more damage to mental health of people. Relative to natural disasters, wars often last longer, and survivors directly exposed to trauma continuously (Morina et al., 2018). Furthermore, the pooled prevalence of PTSS among patients with infectious disease was much higher than healthcare workers, the general population, and suspected cases of infectious diseases, which were consistent with previous studies (Neria et al., 2008). The possible reason is that patients with infectious disease experience higher level of severity of disaster exposure. Patients often directly suffer from the symptoms and traumatic treatment (such as dyspnea, respiratory failure, alteration of conscious states, and tracheotomy), and after being cured, they were more vulnerable to social discrimination than other groups (Neria et al., 2008).

The pooled prevalence of PTSS in different types of diseases was different, and different mortality rates of those infectious diseases also affect the prevalence of PTSS. Among the healthcare workers, mortality rate of infectious diseases was a significant moderator for heterogeneity, higher mortality rate was associated with a higher prevalence of PTSS. Previous studies have shown that when the mortality rate of infectious diseases is high, the impact on mental health of people may be greater (Spoorthy et al., 2020). Therefore, we think the mortality rate of these infectious diseases should be considered when formulating psychological interventions for people influenced by infectious diseases. In addition, the pooled prevalence of PTSS is relatively high in Europe and the Americas, but relatively low in Asia and Africa. The possible reason is that the epidemic situation is more serious in the first two places (WHO, 2020). In addition, the pooled prevalence of PTSS assessed in different time points was different. PTSS among the general population and the healthcare workers were higher in the immediate aftermath of the infectious disease outbreak (0–6 months), which was in line with other studies (Heron-Delaney et al., 2013; Dai et al., 2016; Righy et al., 2019; Benfante et al., 2020). However, in patients with infectious disease, no significant difference was found, and the prevalence of PTSS among patients was still high even after 6 months. This difference in the prevalence estimates among different population may be explained by the fact that patients are exposed to greater trauma than other population, they need more time to recover (Xiao et al., 2020). Furthermore, we found that the pooled prevalence of PTSS among healthcare workers and the general population identified by screening tools was significantly higher than that identified by diagnostic tools, which was consistent with previous researches (Edmondson et al., 2013). It is reported that studies with poor methodological quality or small sample size generally yielded more extreme prevalence estimates (Mata et al., 2015), the current study showed similar results. However, after controlling for other factors, the results of meta-regression showed that the influence of methodological quality and sample size on the prevalence of PTSS is no longer significant. Hence, the results for quality score and sample size in the subgroup analyses require further clarification.

### Implications for the Future

Epidemiological studies have demonstrated a rather high prevalence of mental health problems among different population after an epidemic of infectious disease (Catalan et al., 2011; Tucci et al., 2017). While most of these mental health problems will fade out after the epidemic, symptoms of PTSD may last for a prolonged time and result in severe distress and disability (Vyas et al., 2016). In terms of applicability to COVID-19, evidence suggests that the symptoms of PTSD were very common and persist in patients with infectious disease even higher after 6 months (Hong et al., 2009). Thus, healthcare policies need to take into account both short-term and long-term preventive strategies of PTSD. The information available suggests that the prevalence of PTSS is higher among patients with infectious disease, lower among suspected cases, related workers, and yet even lower in the general population. These three types of samples studied are likely to represent different levels of severity of disaster exposure, with different levels of the PTSS prevalence (Neria et al., 2008). However, there is little doubt that there is a dose–response relationship between the degree of trauma and the mental health burden of disasters (Neria et al., 2008). This relation may not necessarily mean that the principal mental health burden of people exposed to the trauma resulting from infectious disease outbreak is among those who were most directly affected by the disease (Galea and Resnick, 2005). It will be important to establish whether indirect exposure to a trauma during a COVID-19 pandemic was correlated with higher risk of PTSS. In addition, it is necessary to assess the relation between exposure to multiple traumas and risk of PTSS in the future. Additionally, the mortality rate of these infectious diseases should be considered when formulating psychological interventions for people influenced by infectious diseases. Lastly, we think a large multicenter prospective study using a single validated measure of PTSS and measuring possible confounding factors in randomly selected participants is needed in the future, which would provide a more accurate estimate of PTSS among people influenced by infectious diseases.

### Limitations

First, although subgroup analyses and meta-regression analyses were conducted to control many moderating factors for the pooled prevalence of PTSS, heterogeneity was still retained in this review. It is reported that heterogeneity is difficult to avoid in meta-analysis of epidemiological surveys (Winsper et al., 2013), suggesting the need for caution when drawing inferences about estimates of PTSS in post-disaster research. In addition, the follow-up time varies greatly among the included longitudinal studies, which hinder comparability. Additionally, although our study included relevant studies across 30 countries, more than half of the eligible studies were from upper-high income countries. Prevalence studies were scarce for many countries, especially for low-income countries. Considering the inconsistency of the healthcare environment and socioeconomic status across the world, more prevalence studies in low-income countries are needed to understand the panorama of PTSS among people influenced by infectious diseases. Lastly, we noticed that most of the included studies were used screening tools to assess PTSS, only 5.71% of studies used diagnostic tools. It is possible that the pooled prevalence of PTSS caused by infectious diseases was overestimated in this review. Thus, we think ongoing surveillance is essential.

## Conclusion

Evidence suggests that PTSS were very common among people exposed to the trauma resulting from infectious disease outbreak, and the pooled prevalence among different population ranged from 24.20 to 28.83%. Several factors, including type of disease, mortality rate of disease, region, survey time after outbreak, assessment tool, sample size, and quality score, were associated with PTSS. Mortality rate of disease was a significant moderator for heterogeneity. Further research is needed to identify effective strategies for preventing and treating PTSS among people exposed to the trauma resulting from infectious diseases outbreak.

## Data Availability Statement

The original contributions presented in the study are included in the article/Supplementary Material, further inquiries can be directed to the corresponding author/s.

## Author Contributions

DQ, SX, and YL contributed to the design of the study. DQ and YL screened the text. DQ and LL extracted and analyzed the data. JH and FO conducted the quality assessment. DQ wrote the first draft of the manuscript with input from SX. All authors approved the final manuscript.

## Conflict of Interest

The authors declare that the research was conducted in the absence of any commercial or financial relationships that could be construed as a potential conflict of interest.

## Publisher's Note

All claims expressed in this article are solely those of the authors and do not necessarily represent those of their affiliated organizations, or those of the publisher, the editors and the reviewers. Any product that may be evaluated in this article, or claim that may be made by its manufacturer, is not guaranteed or endorsed by the publisher.

## Abbreviations

COVID-19: coronavirus disease 2019
SARS: severe acute respiratory syndrome
MERS-CoV: Middle East respiratory syndrome
Ebola: Ebola virus disease
PTSD: post-traumatic stress disorder
DSM-5: Diagnostic and Statistics of Mental Disorders the fifth edition
H1N1: 2009 influenza A(H1N1)
H7N9: H7N9 avian influenza.
