# Supplementary material for: Infectious Disease Outbreak and Post-Traumatic Stress Symptoms: A Systematic Review and Meta-Analysis
Source: Front Psychol. 2021 Aug 5;12:668784. doi: 10.3389/fpsyg.2021.668784 (PMC8376538; doi:10.3389/fpsyg.2021.668784)
Supplement: Supplementary file 1 [file Data_Sheet_1.PDF]

Supplementary data

Search strategy

Table S1 Search strategy for PubMed:

**a)**	**PTSD**
1.	PTSD [Title/Abstract]
2.	Stress Disorder [Title/Abstract]
3.	Post Traumatic [Title/Abstract]
4.	Posttraumatic [Title/Abstract]
5.	Posttraumatic stress disorder [Title/Abstract]
6.	Post-traumatic stress disorder* [Title/Abstract]
7.	post traumatic syndrome* [Title/Abstract]
8.	posttraumatic syndrome* [Title/Abstract]
9	1 OR 2 OR 3 OR 4 OR 5 OR 6 OR 7 OR 8
**b)**	**COVID-19**
10.	Infection [Title/Abstract]
11.	Infectious [Title/Abstract]
12.	infectious disease [Title/Abstract]
13.	Public Health Event* [Title/Abstract]
14.	public health emergenc* [Title/Abstract]
15.	Flu [Title/Abstract]
16.	Influenza [Title/Abstract]
17.	SARS [Title/Abstract]
18.	Severe Acute Respiratory Syndrome [Title/Abstract]
19.	Ebola [Title/Abstract]
20.	MERS [Title/Abstract]
21.	Middle East Respiratory Syndrome Coronavirus [Title/Abstract]
22.	H1N1 [Title/Abstract]
23.	Zika [Title/Abstract]
24.	Coronavirus [Title/Abstract]
25	COVID-19 [Title/Abstract]
26	10 OR 11 OR 12 OR 13 OR 14 OR 15 OR 16 OR 17 OR 18 OR 19 OR 20 OR 21 OR 22 OR 23 OR 24 OR 25
**c)**	**a) AND b)**
27	9 AND 26 AND

Table S2 The items of quality assessment

Number	Item
A	Is the target population clearly defined?
B	Was either of the following ascertainment methods used [must be one or the other]? (1) probability sampling, or (2) entire population surveyed
C	Is the response rate >70%
D	Are non-responders clearly described?
E	Is the sample representative of the target population (>300 subjects)?
F	Were data collection methods standardized?
G	Were validated criteria used to assess for the presence/absence of disease?
H	Are the estimates of prevalence given with confidence intervals and in detail by subgroup (if applicable)?

Table S3 The results of quality assessment

study	A	B	C	D	E	F	G	H	Total score
Angelina Chan (2004)	1	1	0	0	1	1	1	1	6
Fang Yan (2004)	1	1	1	1	0	1	1	1	7
Laura Hawryluck (2004)	1	0	0	0	0	1	1	1	4
Sim So Sin (2004)	1	1	1	0	0	1	1	1	6
Chengsheng Chen (2005)	1	0	1	0	0	1	1	1	5
Kitty Wu (2005)	1	1	0	0	0	1	0	0	3
Tieying Shi (2005)	1	1	0	0	0	1	0	1	4
Yong Xu (2005)	1	0	1	0	0	1	1	1	5
Zhongguo Liu (2005)	1	1	1	0	0	1	1	0	5
Seow-Khee Kwek (2005)	1	1	0	1	0	1	1	1	6
Hongsheng Gao (2006)	1	1	1	0	0	1	1	0	5
T. M. C. Lee (2006)	1	0	0	0	0	1	1	1	4
Robert G. Maunder (2006)	1	0	0	0	1	1	1	1	5
C-Y Lin (2007)	1	1	1	0	0	1	1	1	6
Laiqi Yang (2007)	1	0	0	0	0	1	1	0	3
TungPing Su (2007)	1	1	0	0	0	1	1	1	5
William J. Lancee (2008)	1	1	0	0	0	1	1	0	4
D.L.Reynolds (2008)	1	1	0	1	1	1	1	1	7
Ping Wu (2008)	1	1	1	0	1	1	1	1	7
Hong Xia (2009)	1	0	1	0	0	1	1	1	5
Ivan Wing Chit Mak (2010)	1	1	1	0	0	1	1	1	6
Kang Sim (2010)	1	0	1	0	1	1	0	1	5
Jiahong Xu (2011)	1	0	1	0	1	1	0	0	4
Charles-Edouard Luyt (2012)	1	0	1	0	0	1	1	1	5
Liling Tang (2016)	1	0	0	0	0	1	0	1	3
Mohamed F Jalloh (2018)	1	1	1	0	1	1	0	1	6

Sang Min Lee (2018)	1	0	0	0	0	1	1	1	4
Heeja Jung (2019)	1	1	0	0	0	1	1	1	5
Lorys Castelli (2020)	1	0	0	0	1	1	0	0	3
Caiyuan Zhang (2020)	1	1	1	0	1	1	1	1	7
Michael Tee (2020)	0	0	1	0	1	1	1	1	5
Mingyu Si (2020)	1	1	1	0	1	1	1	0	6
Rocío Rodríguez-Rey (2020)	1	0	0	0	1	1	1	1	5
Anliu Nie (2020)	1	0	1	0	0	1	1	1	5
Shunwei Liang (2020)	1	1	0	0	1	1	1	1	6
Guo Li (2020)	1	1	1	0	1	1	1	1	7
Emanuele Maria Giusti (2020)	1	1	1	0	1	1	1	0	6
Biao Chen (2020)	1	0	1	0	1	1	1	1	6
Anae¨ lle Caillet (2020)	1	1	0	0	0	1	1	1	5
Mariapaola Barbato (2020)	1	1	0	0	0	1	1	1	5
Abdulmajeed A. Alkhamees (2020)	0	0	0	0	1	1	1	1	4
Yongjie Zhou (2020)	1	0	0	0	1	1	1	1	5
Yuqing Zhao (2020)	0	0	0	0	1	1	0	1	3
Yingfei Zhang (2020)	1	0	0	0	1	1	1	0	4
Qianlan Yin (2020)	1	0	0	0	1	1	1	0	4
U. Wesemann (2020)	1	0	0	0	0	1	0	1	3
Cuiyan Wang (2020)	0	0	1	0	1	1	1	0	4
Mohit Varshney (2020)	0	0	0	0	1	1	1	1	4
Claudia Traunmüller (2020)	1	0	0	0	1	1	1	1	5
Wenjie Tang (2020)	1	1	0	0	1	1	1	1	6
Wanqiu Tan (2020)	1	0	0	0	1	1	1	1	5
Xingyue Song (2020)	1	0	0	0	1	1	1	1	5
Allen C. Sherman (2020)	1	0	0	1	1	1	1	1	6
Emire Seyahi (2020)	1	1	0	0	1	1	1	1	6
Rodolfo Rossi (2020)	1	0	0	0	1	1	0	1	4
Rodolfo Rossi (2020)	1	1	0	0	1	1	1	1	6
Marianna Riello (2020)	1	1	0	0	1	1	1	1	6
Rongfeng Qi (2020)	1	1	0	0	0	1	1	1	5
Zheng Feei Ma (2020)	1	0	1	0	1	1	1	1	6
Lourdes Luceño-Moreno (2020)	1	0	1	0	1	1	1	1	6
Nianqi Liu (2020)	1	0	1	0	0	1	1	0	4
Dong Liu (2020)	1	1	1	0	1	1	0	1	6
Cindy H. Liu (2020)	1	0	0	0	1	1	1	1	5
Yuchen Li (2020)	1	1	1	0	1	1	1	1	7
Yun Li (2020)	0	0	0	0	1	1	1	0	3
Xiuchuan Li (2020)	1	1	1	0	1	1	0	1	6
Xin Li (2020)	1	0	0	0	1	1	1	1	5
Q. Li (2020)	1	0	0	0	1	1	1	1	5
Min Leng (2020)	1	1	1	0	0	1	1	1	6

Xuan Thi Thanh Le (2020)	1	0	0	0	1	1	1	1	5
M. Lange (2020)	1	1	0	0	0	1	1	1	5
Jianbo Lai (2020)	1	1	0	0	1	1	1	1	6
Yael Lahav (2020)	1	0	1	0	1	1	1	0	5
Thanos Karatzias (2020)	1	1	0	0	1	1	1	1	6
Michelle I. Cardel (2020)	1	0	0	0	0	1	1	1	4
Jing Guo (2020)	1	0	0	0	1	1	1	1	5
Clara González-Sanguino (2020)	0	0	0	0	1	1	1	0	3
Leivy Patricia González Ramírez (2020)	1	0	0	0	1	1	1	0	4
Giuseppe Forte (2020)	1	0	0	0	1	1	1	1	5
Feten Fekih-Romdhane (2020)	1	0	0	0	1	1	1	0	4
Safaa M. El-Zoghby (2020)	1	0	0	0	1	1	1	1	5
Hanna Dobson (2020)	1	1	0	0	1	1	1	1	6
Marialaura Di Tella (2020)	1	0	0	0	0	1	0	1	3
Nadia Yanet Cortés-Álvarez (2020)	1	0	1	0	1	1	1	1	6
Alyssa M. Civantos BA (2020)	1	1	0	0	1	1	1	1	6
Alyssa M. Civantos (2020)	1	1	0	0	0	1	1	1	5
Xinli Chi (2020)	1	1	1	0	1	1	0	0	5
Nicholas W. S. Chew (2020)	1	0	1	0	1	1	1	1	6
Min Cheol Chang (2020)	1	1	0	0	0	1	1	1	5
Zhongxiang Cai (2020)	1	0	0	0	1	1	1	1	5
Xin Cai (2020)	1	1	1	0	0	1	0	0	4
Haixin Bo (2020)	1	0	0	0	1	1	1	1	5
Apostolos Blekas (2020)	1	0	0	0	0	1	1	1	4
Yingfei Zhang (2020)	1	1	0	0	0	1	1	1	5
Yaozhi zhang (2020)	1	1	1	0	0	1	1	1	6
Lijun Zhang (2020)	1	0	0	0	0	1	1	1	4
Bo Yuan (2020)	1	1	0	0	0	1	1	0	4
Wenru Xie (2020)	1	0	0	0	0	1	1	1	4
Yanan Liu (2020)	1	0	1	0	1	1	1	1	6
Xianglai Liu (2020)	1	1	1	0	0	1	1	1	6
Fang Leng (2020)	1	0	1	0	0	1	1	0	4
Mei Chen (2020)	1	1	1	0	0	1	1	1	6
Fengyi Hao (2020)	1	1	0	0	0	1	1	1	5
Leilei Liang (2020)	1	0	1	0	1	1	1	1	6
Chuansheng Li (2020)	1	0	1	0	0	1	1	1	5
Jizheng Huang (2020)	1	1	1	0	0	1	1	1	6

MOOSE Checklist

Infectious diseases outbreak and post-traumatic stress symptoms: a systematic review and meta-analysis

Dan Qiu, PhD candidate
Department of Social Medicine and Health Management, Xiangya School of Public Health, Central South University

Yilu Li, PhD candidate
Department of Social Medicine and Health Management, Xiangya School of Public Health, Central South University

Ling Li, PhD candidate
Department of Social Medicine and Health Management, Xiangya School of Public Health, Central South University

Jun He, PhD candidate
Department of Epidemiology and Health Statistics, Xiangya School of Public Health, Central South University

Feiyun Ouyang, PhD candidate
Department of Epidemiology and Health Statistics, Xiangya School of Public Health, Central South University

Shuiyuan Xiao, MD
Department of Social Medicine and Health Management, Xiangya School of Public Health, Central South University;
Department of mental health, Xiangya Hospital, Central South University

Corresponding Author :
Shuiyuan Xiao
Address : Xiangya School of Public Health, Central South University, 110 Xiangya Road, Changsha, HUNAN, 410078, China.
E-mail : shuiyuanxiao1503@163.com
Phone : 86 731 84805454
Fax : 617-566-7805

Criteria		Brief description of how the criteria were handled in the meta-analysis
**Reporting of background should include**		
√	Problem definition	The psychological effects of infectious diseases outbreak can be deleterious and far-reaching. As one of the most widely researched consequence of traumatic events, the prevalence of post-traumatic stress symptoms among people exposed to the trauma resulting from infectious diseases outbreak varies greatly across studies, which remains to be summarized quantitatively.
√	Hypothesis statement	The symptoms of PTSD are very common among people exposed to the trauma resulting from infectious diseases outbreak, relevant study characteristics, such as type of disease, outcome measures, population have an impact on the outcome.
√	Description of study outcomes	prevalence of post-traumatic stress symptoms
√	Type of exposure or intervention used	people exposed to the trauma resulting from infectious diseases outbreak (these outbreaks were SARS, H1N1, H7N9, MERS, Ebola virus disease, Zika virus disease and COVID-19)
√	Type of study designs used	We included case-control studies, prospective cohort studies, cross-sectional studies
√	Study population	people exposed to the trauma resulting from infectious diseases outbreak
**Reporting of search strategy should include**		
√	Qualifications of searchers	The credentials of the two investigators DQ and YLL are indicated in the author list.
√	Search strategy, including time period included in the synthesis and keywords	The following search terms were used: ‘Infectious disease’ (including infectious disease, public health emergency, SARS, H1N1, flu, Ebola, MERS, coronavirus, etc.); ‘Post-traumatic stress disorder’ (including Posttraumatic stress disorder, posttraumatic syndrome, PTSD, stress disorder, etc.). See supplementary data for a full search strategy.<br>We searched with no restrictions on date or language of publication up until 25 April 2020 and an update search was conducted on 14 October 2020.
√	Databases and registries searched	PubMed, EMBASE, Web of Science, the Cochrane Library, PsycArticle, Chinese National Knowledge Infrastructure (CNKI), were independently searched by two reviewers, with no restrictions on date or language of publication up until 25 April 2020 and an update search was conducted on 14 October 2020.

√	Search software used, name and version, including special features	We did not employ a search software. EndNote was used to merge retrieved citations and eliminate duplications
√	Use of hand searching	We hand-searched bibliographies of retrieved papers for additional references
√	List of citations located and those excluded, including justifications	Details of the literature search process are outlined in the flow chart and supplementary data. The citation list is available upon request
√	Method of addressing articles published in languages other than English	We excluded studies not in English and Chinese
√	Method of handling abstracts and unpublished studies	We planned to contacted authors for unpublished studies during the screening process when necessary, no such abstracts and unpublished studies appears in articles that meet the inclusion criteria at last.
√	Description of any contact with authors	Not applicable (All articles that meet the inclusion criteria have complete data for pooled prevalence)
**Reporting of methods should include**		
√	Description of relevance or appropriateness of studies assembled for assessing the hypothesis to be tested	Detailed inclusion and exclusion criteria were described in the methods section.
√	Rationale for the selection and coding of data	Two reviewers (DQ and YLL) checked the titles, abstracts and full-texts of the initial search results independently. Data were extracted on first author, year of publication, country or area, survey period, sample size, response rate, percentage of male participants, average age of participants, instruments used to identify post-traumatic stress symptoms, prevalence of post-traumatic stress symptoms, quality score of the included studies, etc. Any discrepancies that emerged in these procedures were discussed and resolved by involving a third reviewer (SYX).
√	Assessment of confounding	In order to compare the prevalence from different studies (such as survey time after the outbreak, type of disease, diagnostic method, population etc.), we conducted subgroup meta-analysis. The difference between subgroups was examined using the Cochran's Q chi-square tests. Mixed-model meta-regression analyses were performed by using Freeman-Tukey double arcsine method to explore potential moderators on the heterogeneity.
√	Assessment of study quality,	Two independent reviewers (JH and FYOY) used the

	including blinding of quality assessors; stratification or regression on possible predictors of study results	established guidelines, the Loney criteria, to evaluate the methodological quality of the included studies, which has been widely used to evaluate observational studies. The included papers were scored according to eight criteria, such as definition of participants, study design, sampling method, response rate, sample size, appropriateness of measurement and analysis. The scores range from 0 to 8, with a score of 0-3 as low quality, 4-6 as moderate and 7-8 as high. See Table S3 for details on the quality assessment. Publication bias was investigated by funnel plot and Egger's test.
√	Assessment of heterogeneity	Heterogeneity of the studies were explored within two types of study designs using Cochrane's Q test of heterogeneity and I^2 statistic that provides the relative amount of variance of the summary effect due to the between-study heterogeneity.
√	Description of statistical methods in sufficient detail to be replicated	Description of methods of meta-analyses, sensitivity analyses, meta-regression and assessment of publication bias are detailed in the methods.
√	Provision of appropriate tables and graphics	We included 1flow chart,1 summary table, 4 forest plot of all studies, 3 table of subgroup analyses, 1 table of meta regression analysis.<br>In addition, we included 3 supplementary tables in the supplementary data file.
**Reporting of results should include**		
√	Graph summarizing individual study estimates and overall estimate	Figure 2
√	Table giving descriptive information for each study included	Table 1
√	Results of sensitivity testing	Table S3
√	Indication of statistical uncertainty of findings	95% confidence intervals were presented with all summary estimates, I^2 values and results of sensitivity analyses
**Reporting of discussion should include**		
√	Quantitative assessment of bias	The results of the Egger's test showed that publication bias was not found in this study and the sensitivity analysis showed that no individual study significantly influenced the overall results. However, the observed heterogeneity should be noticed.
√	Justification for exclusion	We excluded studies that not write in English or Chinese,

		which was a limitation in this review.
√	Assessment of quality of included studies	We discussed the results of the subgroup analyses, and potential reasons for the observed heterogeneity.
**Reporting of conclusions should include**		
√	Consideration of alternative explanations for observed results	We noted that the variations in the prevalence may be due to true population differences, or to differences in quality of studies, survey time, etc.
√	Generalization of the conclusions	Evidence suggests that the symptoms of PTSD were very common among people exposed to the trauma resulting from infectious diseases outbreak and may last for a prolonged time. Healthcare policies need to take into account both short-term and long-term preventive strategy of PTSD in the forthcoming months.
√	Guidelines for future research	At first, healthcare policies need to take into account both short-term and long-term preventive strategy of PTSD in the forthcoming months. It will be important to establish whether indirect exposure to a trauma during a COVID-19 pandemic was correlated with higher risk of PTSD. Also, it is necessary to assess the relation between exposure to multiple traumas and risk of PTSD in the future. Additionally, we think a large multicenter prospective study using a single validated measure of PTSD and measuring possible confounding factors in randomly selected participants is needed in the future, which would provide a more accurate estimate of PTSD among patients of COVID-19. At last, we think ongoing surveillance is essential.
√	Disclosure of funding source	This research was supported by the Ministry of Science and Technology of China (Grant NO: 2016YFC0900802). The funding agency did not take part in the design of the study and collection, analysis, and interpretation of data and in writing the manuscript.

Section / topic	#	Checklist item	Reported on page #
**TITLE**			
Title	1	Infectious diseases outbreak and post-traumatic stress symptoms: a systematic review and meta-analysis	Title
**ABSTRACT**			
Structured summary	2	Abstract<br><br>Background: As one of the most widely researched consequence of traumatic events, the prevalence of post-traumatic stress symptoms among people exposed to the trauma resulting from infectious diseases outbreak varies greatly across studies. This review aimed at examining the pooled prevalence of post-traumatic stress symptoms among people exposed to the trauma resulting from infectious diseases outbreak, summarizing the possible causes of the inconsistencies in the current estimates.<br><br>Methods: Systematic searches of databases were conducted for literature published on PubMed, EMBASE, Web of Science, the Cochrane Library, PsycArticle, CNKI until 14 October 2020. Statistical analyses were performed using R software.<br><br>Results: Of 106 studies were included. The results showed that the pooled prevalence of post-traumatic stress symptoms among the general population exposed to the trauma resulting from infectious diseases outbreak was 24.20% (95% CI: 18.54% to 30.53%), the pooled prevalence of post-traumatic stress symptoms among healthcare workers was 24.35% (95% CI: 18.38% to 31.51%), the pooled prevalence of post-traumatic stress symptoms among the patients of infectious disease was 28.83% (95% CI: 18.53% to 44.86%), and the pooled prevalence of post-traumatic stress symptoms among suspected cases of infectious disease was 25.04% (95% CI: 18.05% to 34.73%). Mortality rate was a significant contributor to heterogeneity.<br><br>Conclusions: Evidence suggests that post-traumatic stress symptoms were very common among people exposed to the trauma resulting from infectious diseases outbreak. Health policymakers should consider both short-term and long-term preventive strategy of post-traumatic stress symptoms.	Abstract
**INTRODUCTION**			

Rationale	3	Over the past 2 decades, novel viruses continuing to emerge, such as SARS in 2003, H1N1 in 2009, Ebola in 2014. The outbreak of infectious diseases can spread rapidly, causing enormous losses to individual health, national economy, and social wellbeing. The psychological effects of infectious diseases outbreak can be deleterious and far-reaching. As one of the most widely researched consequence of traumatic events, the prevalence of post-traumatic stress symptoms among people exposed to the trauma resulting from infectious diseases outbreak varies greatly across studies. For taking effective measures to reduce the psychological sequelae caused by infectious diseases outbreak across the world, determine a more accurate estimation of the prevalence of post-traumatic stress symptoms, understanding how infectious diseases outbreak cause PTSD and who might be vulnerable are essential.	Introduction
Objectives	4	This review aimed at examining the pooled prevalence of post-traumatic stress symptoms among people exposed to the trauma resulting from infectious diseases outbreak, summarizing possible vulnerability factors of post-traumatic stress symptoms and examining potentially vulnerable populations, try to provide a reference for COVID-19 and possible outbreak of infectious diseases in the future.	Introduction
**METHODS**			
Protocol and registration	5	This review was reported in accordance with the PRISMA guideline and MOOSE guidelines. The protocol of this review is registered in the International Prospective Register of Systematic Reviews (registration number: CRD42020182366).	Methods
Eligibility criteria	6	Studies were included if they meet the following criteria: (1) the study was observational study; (2) information about prevalence of post-traumatic stress symptoms among people exposed to the trauma resulting from infectious diseases outbreak of PTSD was provided; (3) the full article was written in English or Chinese; (4) these outbreaks were SARS, H1N1, H7N9, MERS, Ebola virus disease, Zika virus disease and COVID-19. Studies were excluded if: (1) the report was a review, comments, meta-analysis or protocol; (2) the participants with comorbid symptoms or chronic disease (such as mental illness, cancer, etc.); (3) the report was duplicate results.	Methods
Information sources	7	PubMed, EMBASE, Web of Science, the Cochrane Library, PsycArticle, Chinese National Knowledge Infrastructure (CNKI), were independently searched by two reviewers, with no restrictions on date or language of publication up until 25 April 2020 and an update search was conducted on 14 October 2020. The following search terms were used: 'Infectious disease' (including infectious disease, public health emergency, SARS, H1N1, flu, Ebola, MERS, coronavirus, etc.); 'Post-traumatic stress disorder' (including	Methods

		Posttraumatic stress disorder, posttraumatic syndrome, PTSD, stress disorder, etc.). See supplementary data for a full search strategy.	
Search	8	**A. PTSD**<br>1. PTSD **[Title/Abstract]**<br>2. Stress Disorder **[Title/Abstract]**<br>3. Post Traumatic **[Title/Abstract]**<br>4. Posttraumatic **[Title/Abstract]**<br>5. Posttraumatic stress disorder **[Title/Abstract]**<br>6. Post-traumatic stress disorder* **[Title/Abstract]**<br>7. post traumatic syndrome* **[Title/Abstract]**<br>8. posttraumatic syndrome* **[Title/Abstract]**<br>9. 1 OR 2 OR 3 OR 4 OR 5 OR 6 OR 7 OR 8<br><br>**B. infectious disease**<br>10. Infection **[Title/Abstract]**<br>11. Infectious **[Title/Abstract]**<br>12. infectious disease **[Title/Abstract]**<br>13. Public Health Event* **[Title/Abstract]**<br>14. public health emergenc* **[Title/Abstract]**<br>15. Flu **[Title/Abstract]**<br>16. Influenza **[Title/Abstract]**<br>17. SARS **[Title/Abstract]**<br>18. Severe Acute Respiratory Syndrome **[Title/Abstract]**<br>19. Ebola **[Title/Abstract]**<br>20. MERS **[Title/Abstract]**<br>21. Middle East Respiratory Syndrome Coronavirus **[Title/Abstract]**<br>22. H1N1 **[Title/Abstract]**<br>23. Zika **[Title/Abstract]**<br>24. Coronavirus **[Title/Abstract]**<br>25. 10 OR 11 OR 12 OR 13 OR 14 OR 15 OR 16 OR 17 OR 18 OR 19 OR 20 OR 21 OR 22 OR 23 OR 24<br>26. 9 AND 25	Supplementary data
Study selection	9	Two reviewers (DQ and YLL) checked the titles, abstracts and full-texts of the initial search results independently. Data were extracted on first author, year of publication, country or area, survey period, sample size, response rate, percentage of male participants, average age of participants, instruments used to identify post-traumatic stress symptoms, prevalence of post-traumatic stress symptoms, quality score of the included studies, etc. Any discrepancies that emerged in these procedures were discussed and resolved by involving a third reviewer (SYX).	Methods
Data collection process	10	Two reviewers (DQ and YLL) checked the titles, abstracts and full-texts of the initial search results independently. Any discrepancies that	Methods

		emerged in these procedures were discussed and resolved by involving a third reviewer (SYX).	
Data items	11	Data were extracted on first author, year of publication, country or area, survey period, sample size, response rate, percentage of male participants, average age of participants, instruments used to identify post-traumatic stress symptoms, prevalence of post-traumatic stress symptoms, quality score of the included studies, etc.	Methods
Risk of bias in individual studies	12	Two independent reviewers (JH and FYOY) used the established guidelines, the Loney criteria, to evaluate the methodological quality of the included studies, which has been widely used to evaluate observational studies. The included papers were scored according to eight criteria, such as definition of participants, study design, sampling method, response rate, sample size, appropriateness of measurement and analysis. The scores range from 0 to 8, with a score of 0-3 as low quality, 4-6 as moderate and 7-8 as high. See Table S3 for details on the quality assessment.	Methods
Summary measures	13	prevalence of post-traumatic stress symptoms	Methods
Synthesis of results	14	When data were available for three or more studies, prevalence or risk factor was combined. When there were 10 or more studies, quantitative subgroup analysis was conducted. All the statistical analyses were performed using the "meta" (4.12-0) and "metafor" package (2.4-0) of R version 4.0.0. Between-study heterogeneity was evaluated by Cochran's Q test and quantified by the I^2 statistic, with values 50% or more indicating possible heterogeneity. The pooled prevalence of post-traumatic stress symptoms was combined using Logit transformation method or Log transformation method by a random effects model if significant heterogeneity was observed across studies (when $P < 0.05$, I2 > 50%). If more than one dataset was reported for the same group of participants, the outcomes that were assessed at the baseline were used.	Methods

**Section/topic**	**#**	**Checklist item**	**Reported on page #**
Risk of bias across studies	15	Publication bias was investigated by Egger's test. To evaluate the consistency of the results, sensitivity analysis was performed by removing each study individually. All the statistical tests were 2-sided, with a significance threshold of $P < 0.05$.	Methods
Additional analyses	16	In order to compare the prevalence from different studies (such as survey time after the outbreak, type of disease, diagnostic method, population etc.), we conducted subgroup meta-analysis. The difference between subgroups was examined using the Cochran's Q chi-square tests. Mixed-model meta-regression analyses were performed by using	Methods

		Freeman-Tukey double arcsine method to explore potential moderators on the heterogeneity.	
**RESULTS**			
Study selection	17	As shown in Fig. 1, a total of 6612 references were identified. Among them, 2953 duplicates were removed. By screening titles and abstracts, 3019 irrelevant articles were excluded. A total of 288 potentially relevant full-text articles were independently assessed based on the selection criteria. Further, 183 studies were excluded because of the following reasons: duplicate articles or results (n = 15); review (n = 1); did not provide data on PTSD (n = 115); not infectious disease (n = 44); unable to locate full text (n = 7); not in English or Chinese (n = 1). Finally, 106 eligible studies were included in this review. See Fig. 1 for the details.	Results
Study characteristics	18	One hundred and five papers met the inclusion criteria. Of the included studies, seventy-seven were of COVID-19, (21-97) two of MERS, (11, 98) one of Ebola virus disease, (99) one of H7N9, (100) two of H1N1, (101, 102) and the remaining 22 of SARS (10, 103-123). Six papers were in Chinese, and the remainder in English. Of these, ninety- two were cross sectional studies, nine were longitudinal designs and four were case control studies. Most of the included studies were from Asia, such as China, Singapore and South Korea. See Table 1 for the details.	Results
Risk of bias within studies	19	From the 106 papers, five (4.72%) studies were rated as high quality, 93 (87.73%) were rated as moderate, and eight (7.55%) were rated as low quality. Details of the methodological quality assessments of all 106 studies are showed in Table S3.	Results
Results of individual studies	20	There were 51 studies reported prevalence of post-traumatic stress symptoms among the general population. The forest plot in Fig. 2 depicts the details. A total of 78459 people exposed to the trauma resulting from an epidemic of infectious disease were identified in the 51 articles, of which 25826 were reported with post-traumatic stress symptoms. The random effects model was used to determine the pooled prevalence (I^2 = 99.80%, $P < 0.001$), the pooled prevalence of post-traumatic stress symptoms among people exposed to the trauma resulting from infectious diseases outbreak was 24.20%, with a 95% CI of 18.54% to 30.53%.<br>A total of 41 studies reported prevalence of post-traumatic stress symptoms among the healthcare workers. The forest plot in Fig. 3 depicts the details. A total of 38250 healthcare workers exposed to the trauma resulting from an epidemic of infectious disease were identified in the 41 articles, of which 9071 were reported with post-traumatic stress symptoms. The random effects model was used to determine the pooled prevalence (I^2 = 99.40%, $P < 0.001$), the pooled prevalence of post-traumatic stress symptoms among healthcare workers exposed to the trauma resulting from infectious diseases outbreak was 24.35%,	Results

		with a 95% CI of 18.38% to 31.51%.<br>A total of 15 studies reported prevalence of post-traumatic stress symptoms among the patients. The forest plot in Fig. 4 depicts the details. A total of 2666 patients exposed to the trauma resulting from an epidemic of infectious disease were identified in the 15 articles, of which 1125 were reported with post-traumatic stress symptoms. The random effects model was used to determine the pooled prevalence (I2 = 98.60%, $P < 0.001$), the pooled prevalence of post-traumatic stress symptoms among patients exposed to the trauma resulting from infectious diseases outbreak was 28.83%, with a 95% CI of 18.53% to 44.86%.<br>A total of 3 studies reported prevalence of post-traumatic stress symptoms among the suspected cases. The forest plot in Fig. 5 depicts the details. A total of 525 suspected cases of infectious disease exposed to the trauma resulting from an epidemic of infectious disease were identified in the 3 articles, of which 139 were reported with post-traumatic stress symptoms. The random effects model was used to determine the pooled prevalence (I2 = 74.50%, $P < 0.001$), the pooled prevalence of post-traumatic stress symptoms among suspected cases exposed to the trauma resulting from infectious diseases outbreak was 25.04%, with a 95% CI of 18.05% to 34.73%.	
Synthesis of results	21	There were 51 studies reported prevalence of post-traumatic stress symptoms among the general population. The forest plot in Fig. 2 depicts the details. A total of 78459 people exposed to the trauma resulting from an epidemic of infectious disease were identified in the 51 articles, of which 25826 were reported with post-traumatic stress symptoms. The random effects model was used to determine the pooled prevalence (I2 = 99.80%, $P < 0.001$), the pooled prevalence of post-traumatic stress symptoms among people exposed to the trauma resulting from infectious diseases outbreak was 24.20%, with a 95% CI of 18.54% to 30.53%.<br>A total of 41 studies reported prevalence of post-traumatic stress symptoms among the healthcare workers. The forest plot in Fig. 3 depicts the details. A total of 38250 healthcare workers exposed to the trauma resulting from an epidemic of infectious disease were identified in the 41 articles, of which 9071 were reported with post-traumatic stress symptoms. The random effects model was used to determine the pooled prevalence (I2 = 99.40%, $P < 0.001$), the pooled prevalence of post-traumatic stress symptoms among healthcare workers exposed to the trauma resulting from infectious diseases outbreak was 24.35%, with a 95% CI of 18.38% to 31.51%.<br>A total of 15 studies reported prevalence of post-traumatic stress symptoms among the patients. The forest plot in Fig. 4 depicts the details. A total of 2666 healthcare workers exposed to the trauma	Results

		resulting from an epidemic of infectious disease were identified in the 15 articles, of which 1125 were reported with post-traumatic stress symptoms. The random effects model was used to determine the pooled prevalence (I2 = 98.60%, P < 0.001), the pooled prevalence of post-traumatic stress symptoms among patients exposed to the trauma resulting from infectious diseases outbreak was 28.83%, with a 95% CI of 18.53% to 44.86%.<br>A total of 3 studies reported prevalence of post-traumatic stress symptoms among the suspected cases. The forest plot in Fig. 5 depicts the details. A total of 525 suspected cases of infectious disease exposed to the trauma resulting from an epidemic of infectious disease were identified in the 3 articles, of which 139 were reported with post-traumatic stress symptoms. The random effects model was used to determine the pooled prevalence (I2 = 74.50%, P < 0.001), the pooled prevalence of post-traumatic stress symptoms among suspected cases exposed to the trauma resulting from infectious diseases outbreak was 25.04%, with a 95% CI of 18.05% to 34.73%.	
Risk of bias across studies	22	The results of the Egger's test showed that publication bias was not found in this study (t = -2.425, p = 0.208). When each study was excluded one-by-one, the recalculated combined results did not change significantly. The pooled prevalence of post-traumatic stress symptoms ranged from 23.36% (95% CI: 17.87-29.92%) to 25.34% (95% CI: 19.55-32.16%), and the I2 statistic varied from 99.70% to 99.80%. The results indicate that no individual study significantly influenced the overall results.<br>The results the Egger's test showed that publication bias was not found in this study (t =0.728, p = 0.470). When each study was excluded one-by-one, the recalculated combined results did not change significantly. The pooled prevalence of post-traumatic stress symptoms ranged from 23.22% (95% CI: 17.69-29.84%) to 25.62% (95% CI: 19.68-32.62%), and the I2 statistic varied from 99.20% to 99.40%. The results indicate that no individual study significantly influenced the overall results.<br>The results of the Egger's test showed that publication bias was not found in this study (t =-6.138, p = 3.553). When each study was excluded one-by-one, the recalculated combined results did not change significantly. The pooled prevalence of post-traumatic stress symptoms ranged from 23.22% (95% CI: 17.69-29.84%) to 32.23% (95% CI: 20.75-50.05%), and the I2 statistic varied from 95.40% to 98.7%. The results indicate that no individual study significantly influenced the overall results.	Results
Additional analysis	23	The details of subgroup analyses are presented in Table 2. There were no significant differences in the prevalence of post-traumatic stress symptoms between age and gender (Q=0.08 and 0.16, P > 0.05). Significant difference in the prevalence of post-traumatic stress	Results

		symptoms between different type of disease was observed, the pooled prevalence of post-traumatic stress symptoms among people influenced by COVID-19 was higher than that for people influenced by SARS, Ebola and H1N1 (26.75% vs. 16.42% vs. 15.99% vs. 2.03%; Q= 117.12, $P < 0.05$). Also, a higher Mortality rate is associated with a lower prevalence of PTSS (24.39% vs. 15.99%; Q= 8.26, $P < 0.05$). The pooled prevalence of post-traumatic stress symptoms among people in the Eastern Mediterranean region was higher than people in the Western Pacific region, the South-East Asia region, the America region, the European region and the Africa region (37.74% vs. 33.23% vs. 29.25% vs. 24.00% vs. 20.78% vs. 15.99%; Q= 114.16, $P < 0.05$). Furthermore, there were significant differences in prevalence of post-traumatic stress symptoms between different survey time after the outbreak, closer survey time to the point of infectious disease outbreak was associated with higher prevalence of post-traumatic stress symptoms (25.96% vs. 5.95%; Q= 7.49, $P < 0.05$). There were significant differences in the prevalence of post-traumatic stress symptoms between studies used different assessment tools (24.44% vs. 14.00%; Q= 12.18, $P < 0.05$). In addition, significant difference in the prevalence of post-traumatic stress symptoms between studies with different quality scores was observed, articles with the highest quality scores showed high prevalence (12.57% vs. 12.41% vs. 25.86%; Q= 19.00, $P < 0.05$). A multivariate meta-regression was carried out to explore the origin of heterogeneity accounted for by the variables such as type of disease, survey time after the outbreak. However, no significant contributor was found. see Table 5 for the details.<br>The details of subgroup analyses are presented in Table 3. There were no significant differences in the prevalence of post-traumatic stress symptoms between age, gender, mortality rate of disease, sample size and quality score (Q=0.21, 0.19, 3.78, 2.54 and 4.65, $P > 0.05$). Significant difference in the prevalence of post-traumatic stress symptoms between different type of disease was observed, the pooled prevalence of post-traumatic stress symptoms among people influenced by MERS was higher than that for people influenced by COVID-19, H7N9 and SARS (52.77% vs. 29.64% vs. 20.59% vs. 11.80%; Q= 351.95, $P < 0.05$). Also, a higher mortality rate is associated with a higher prevalence of post-traumatic stress symptoms (23.19% vs. 42.04%; Q= 3.78, $P < 0.05$). The pooled prevalence of post-traumatic stress symptoms among people in the European region was higher than people in the America region, the Western Pacific region and the South-East Asia region (34.47% vs. 29.10% vs. 21.70% vs. 7.94%; Q= 70.59, $P < 0.05$). Furthermore, there were significant differences in prevalence of post-traumatic stress symptoms between different survey time after the outbreak, closer survey time to the point of infectious disease	

		outbreak are associated with higher prevalence of post-traumatic stress symptoms (29.04% vs. 10.42%; Q= 10.09, $P < 0.05$). There were significant differences in the prevalence of post-traumatic stress symptoms between studies used different assessment tools (24.87% vs. 8.93%; Q= 5.84, $P < 0.05$). A multivariate meta-regression was carried out to explore the origin of heterogeneity accounted for by the variables including type of disease, mortality rate, survey time after the outbreak, age, gender, quality score and sample size. The results of meta-regression showed that mortality rate of disease was a significant contributor to heterogeneity (accounted for 16.81% of the heterogeneity). see Table 5 for the details.<br>The details of subgroup analyses are presented in Table 4. There were no significant differences in the prevalence of post-traumatic stress symptoms between age, gender, type of disease, region, survey time after outbreak, diagnosis tool, sample size and quality score ($P > 0.05$). Significant difference in the prevalence of post-traumatic stress symptoms between studies with different quality scores was observed (5.64% vs. 35.45% vs. 9.79%; Q= 31.65, $P < 0.05$). A multivariate meta-regression was carried out to explore the origin of heterogeneity accounted for by the variables such as type of disease, survey time after the outbreak. However, no significant contributor was found. see Table 5 for the details.	
**DISCUSSION**			
Summary of evidence	24	This review has highlighted the importance of considering the psychological impacts of people exposed to the trauma resulting from infectious diseases outbreak. The results showed that the pooled prevalence of PTSD symptoms among the general population exposed to the trauma resulting from infectious diseases outbreak was 24.20% (95% CI: 18.54% to 30.53%), the pooled prevalence of PTSD symptoms among the healthcare workers exposed to the trauma resulting from infectious diseases outbreak was 24.35% (95% CI: 18.38% to 31.51%), the pooled prevalence of PTSD symptoms among the patients of infectious disease was 28.83% (95% CI: 18.53% to 44.86%), and the pooled prevalence of PTSD symptoms among the suspected cases of infectious disease was 25.04% (95% CI: 18.05% to 34.73%), and mortality rate of disease was associated with PTSD symptoms.	Discussion
Limitations	25	Firstly, we excluded studies were not written in English or Chinese. Besides, although subgroup analyses and meta-regression analyses were conducted to control many moderating factors for the pooled prevalence of PTSD symptoms, heterogeneity was still remained in this review. It is reported that heterogeneity is difficult to avoid in meta-analysis of epidemiological surveys,(138) which suggesting the need for caution when drawing inferences about estimates of PTSD in post-	Discussion

		disaster research. Also, the follow-up time varies greatly among the included longitudinal studies, which hinders comparability. Additionally, although our study included relevant studies across 30 countries, more than half of the eligible studies were from upper-high income countries. Prevalence studies were scarce for many countries, especially for low-income countries. Considering the inconsistency of the health care environment and socioeconomic status across the world, more prevalence studies in low-income countries are needed to understand the panorama of PTSD among people influenced by infectious diseases. Lastly, we noticed that most of the included studies were used screening tools to assess PTSD symptoms, only 5.71% of studies used diagnostic tools. It is possible that the pooled prevalence of PTSD symptoms caused by infectious diseases was overestimated in this review. Thus, we think ongoing surveillance is essential.	
Conclusions	26	Evidence suggests that the symptoms of PTSD were very common among people exposed to the trauma resulting from infectious diseases outbreak and may last for a prolonged time. Healthcare policies need to take into account both short-term and long-term preventive strategy of PTSD in the forthcoming months.	Discussion
**FUNDING**			
Funding	27	This research was supported by the Ministry of Science and Technology of China (Grant NO: 2016YFC0900802).	Funding

From: Moher D, Liberati A, Tetzlaff J, Altman DG, The PRISMA Group (2009). Preferred Reporting Items for Systematic Reviews and Meta-Analyses: The PRISMA Statement. PLoS Med 6(6): e1000097. doi:10.1371/journal.pmed1000097

For more information, visit: **www.prisma-statement.org**.
